# Marine Pharmacology in 2012–2013: Marine Compounds with Antibacterial, Antidiabetic, Antifungal, Anti-Inflammatory, Antiprotozoal, Antituberculosis, and Antiviral Activities; Affecting the Immune and Nervous Systems, and Other Miscellaneous Mechanisms of Action †

**DOI:** 10.3390/md15090273

**Published:** 2017-08-29

**Authors:** Alejandro M. S. Mayer, Abimael D. Rodríguez, Orazio Taglialatela-Scafati, Nobuhiro Fusetani

**Affiliations:** 1 Department of Pharmacology, Chicago College of Osteopathic Medicine, Midwestern University, 555 31st Street, Downers Grove, IL 60515, USA; 2 Molecular Sciences Research Center, University of Puerto Rico, 1390 Ponce de León Avenue, San Juan, PR 00926, USA; abimael.rodriguez1@upr.edu; 3 Department of Pharmacy, University of Naples “Federico II”, Via D. Montesano 49, 80131 Napoli, Italy; scatagli@unina.it; 4 Fisheries and Oceans Hakodate, Hakodate 041-8611, Japan; anobu@fish.hokudai.ac.jp

**Keywords:** drug, marine, chemical, metabolite, natural product, pharmacology, pharmaceutical, review, toxicology, pipeline

## Abstract

The peer-reviewed marine pharmacology literature from 2012 to 2013 was systematically reviewed, consistent with the 1998–2011 reviews of this series. Marine pharmacology research from 2012 to 2013, conducted by scientists from 42 countries in addition to the United States, reported findings on the preclinical pharmacology of 257 marine compounds. The preclinical pharmacology of compounds isolated from marine organisms revealed antibacterial, antifungal, antiprotozoal, antituberculosis, antiviral and anthelmitic pharmacological activities for 113 marine natural products. In addition, 75 marine compounds were reported to have antidiabetic and anti-inflammatory activities and affect the immune and nervous system. Finally, 69 marine compounds were shown to display miscellaneous mechanisms of action which could contribute to novel pharmacological classes. Thus, in 2012–2013, the *preclinical* marine natural product pharmacology pipeline provided novel pharmacology and lead compounds to the *clinical* marine pharmaceutical pipeline, and contributed significantly to potentially novel therapeutic approaches to several global disease categories.

## 1. Introduction

**Figure 1:**
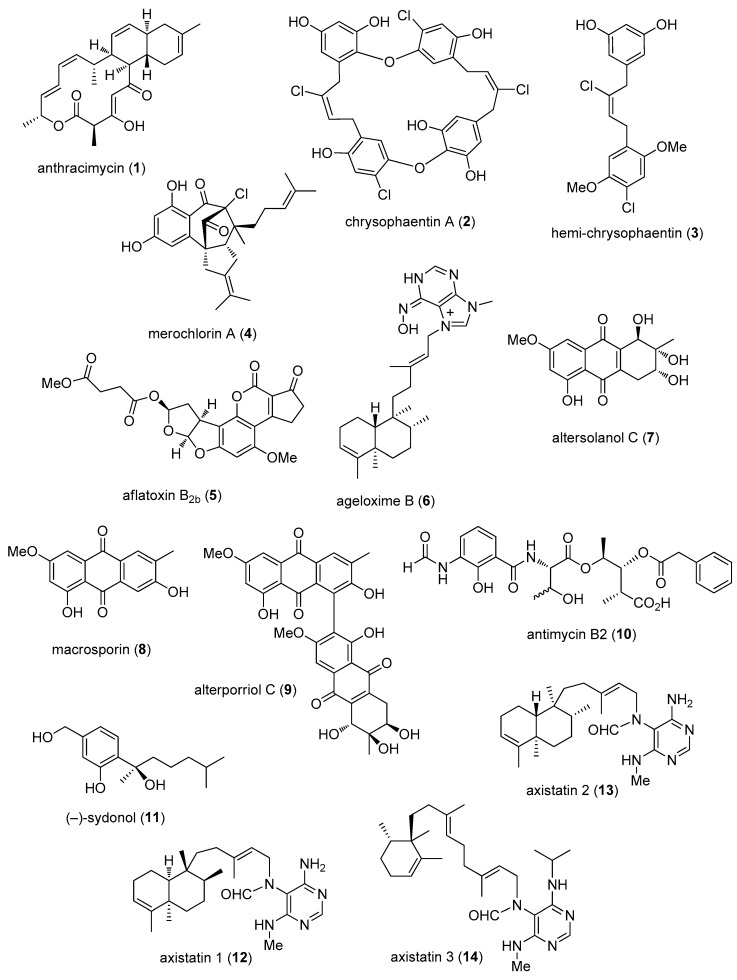

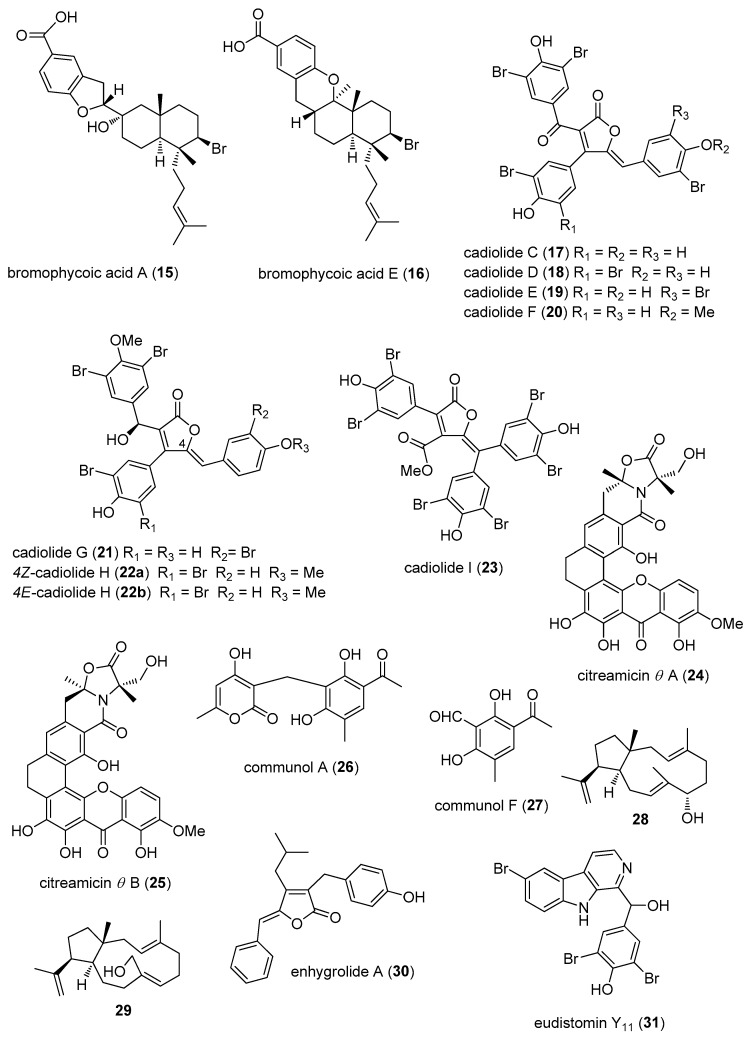

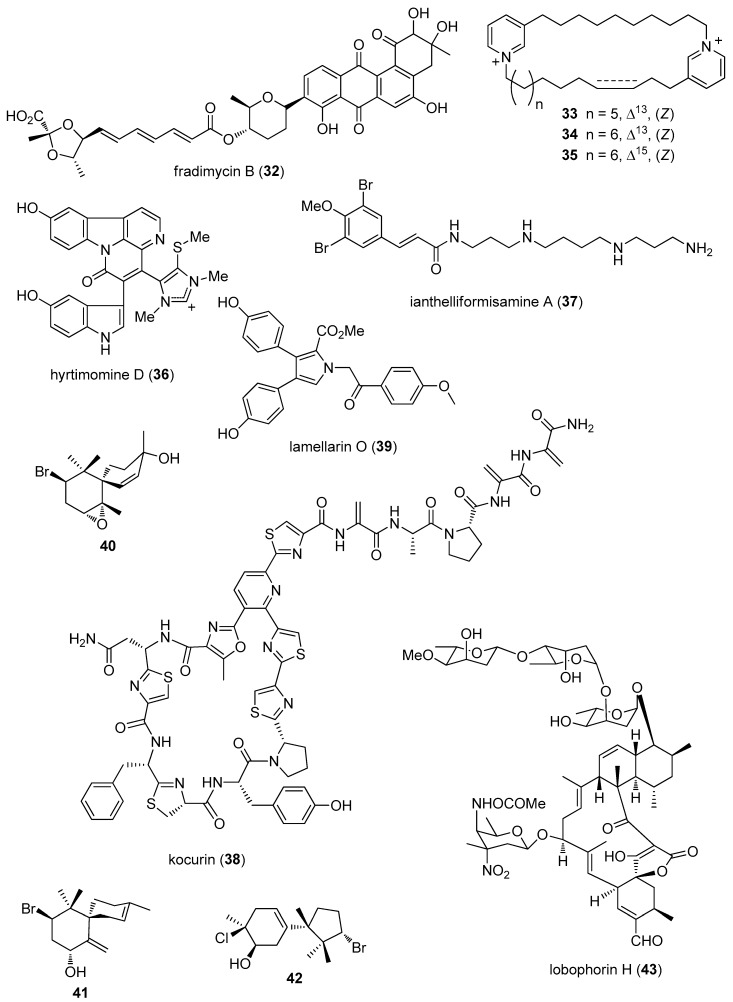

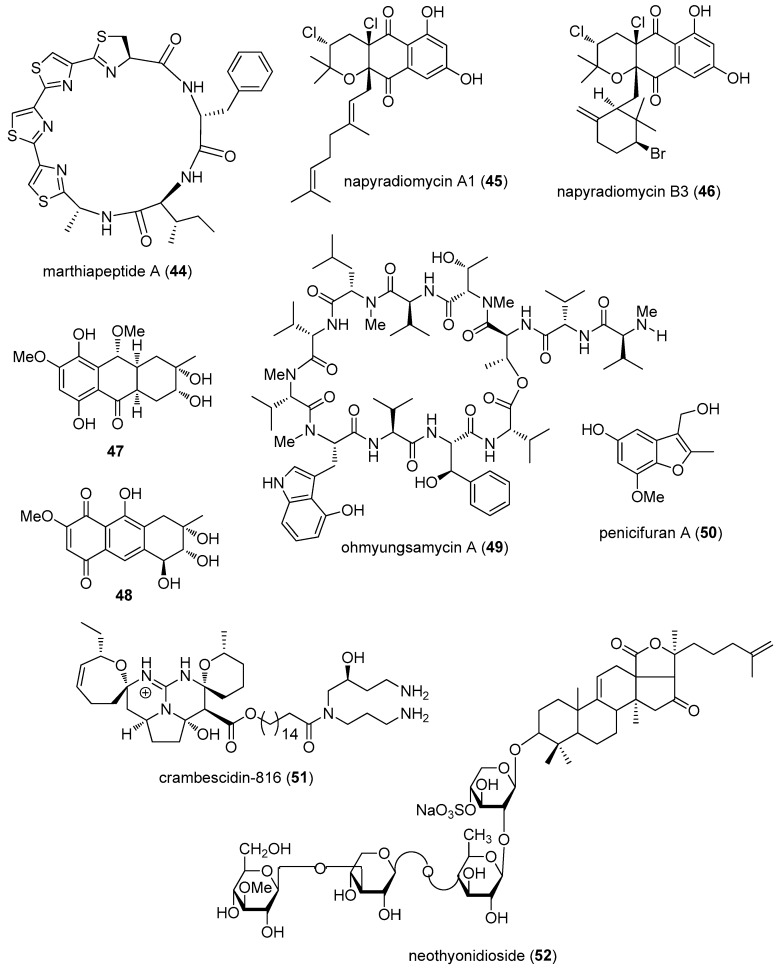

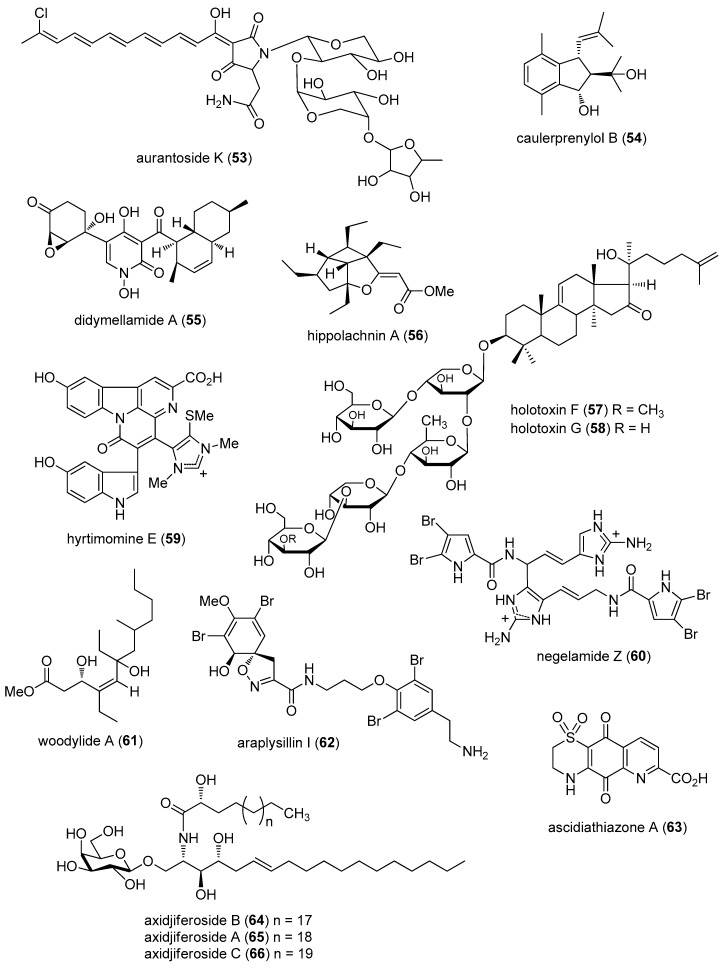

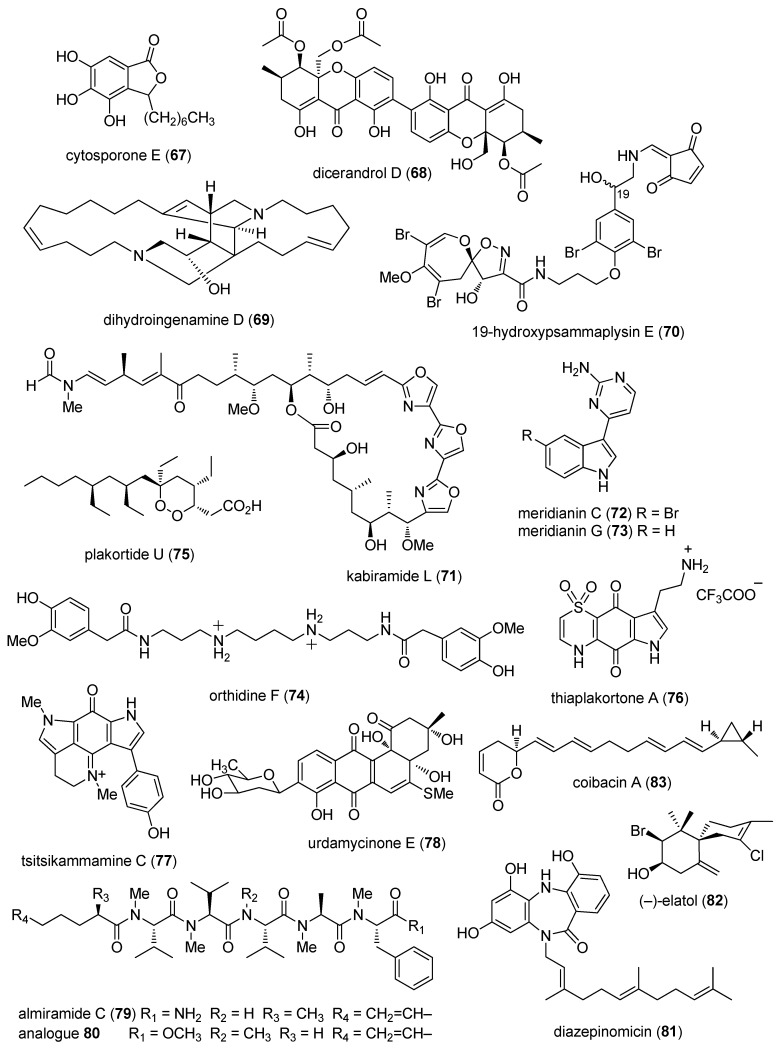

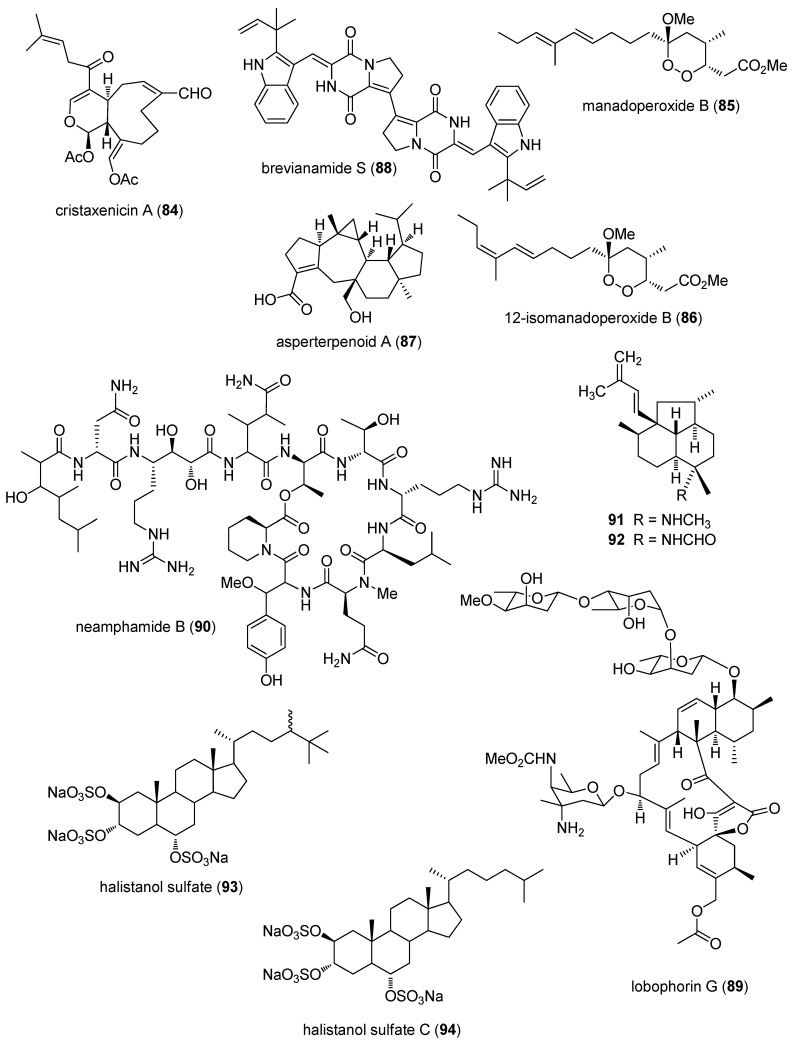

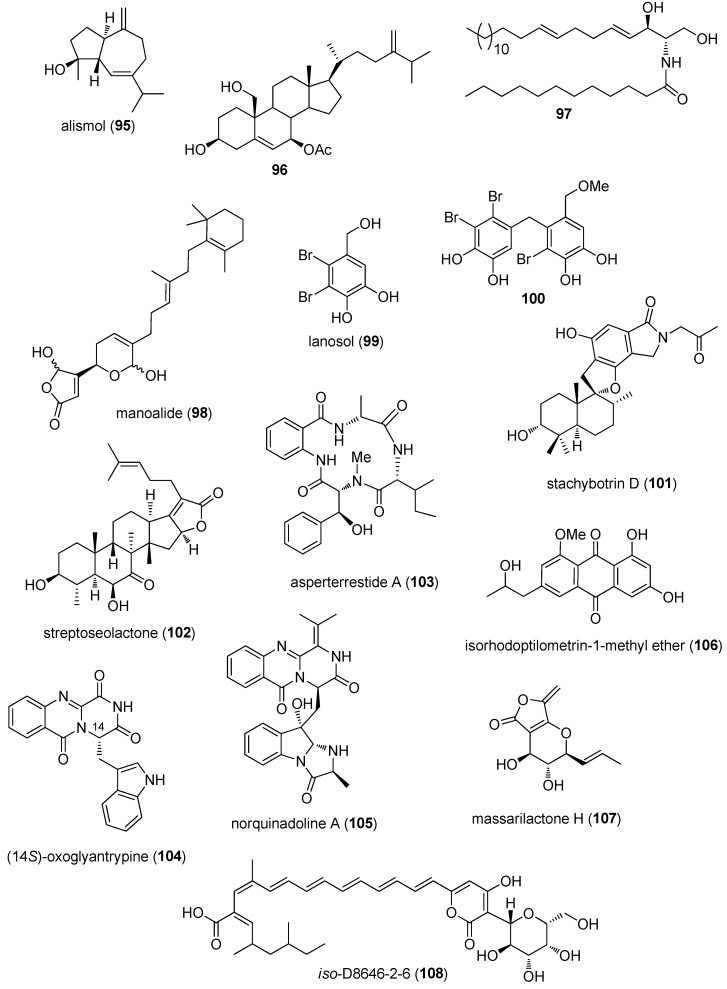

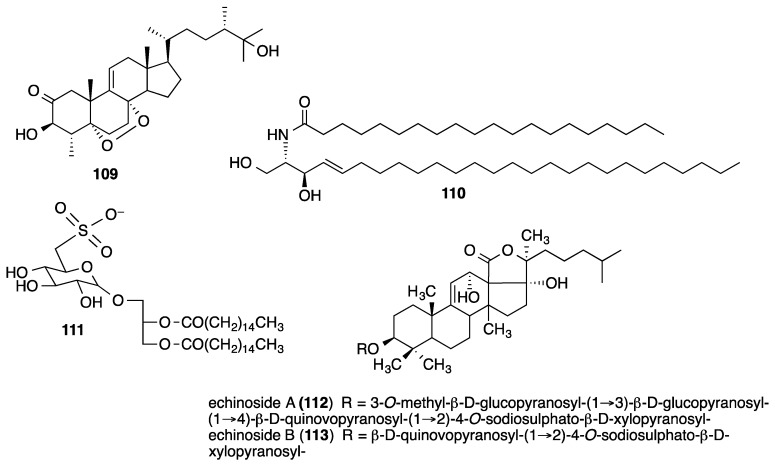
Marine pharmacology in 2012–2013: marine compounds with antibacterial, antifungal, antiprotozoal, antituberculosis, and antiviral activities.

**Figure 2:**
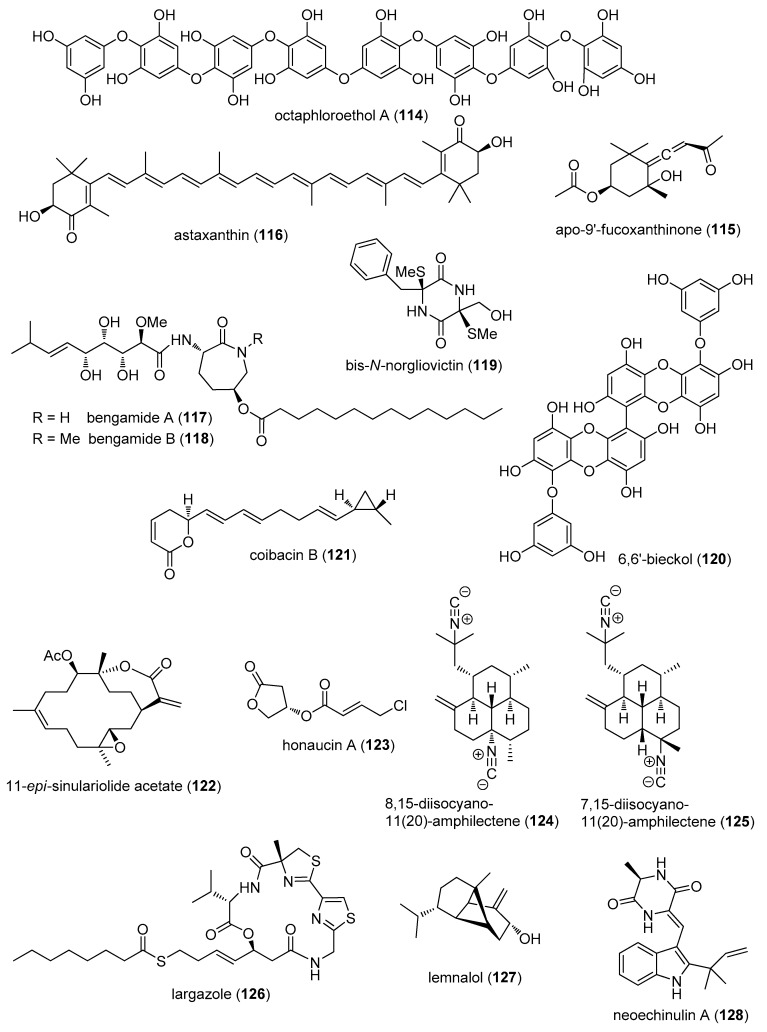

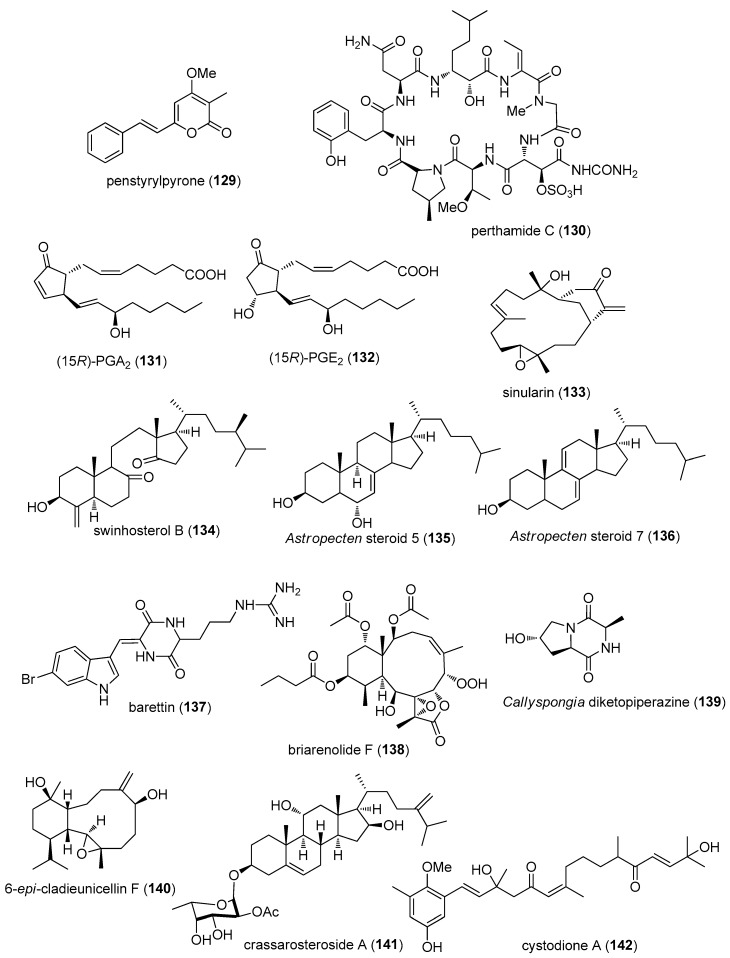

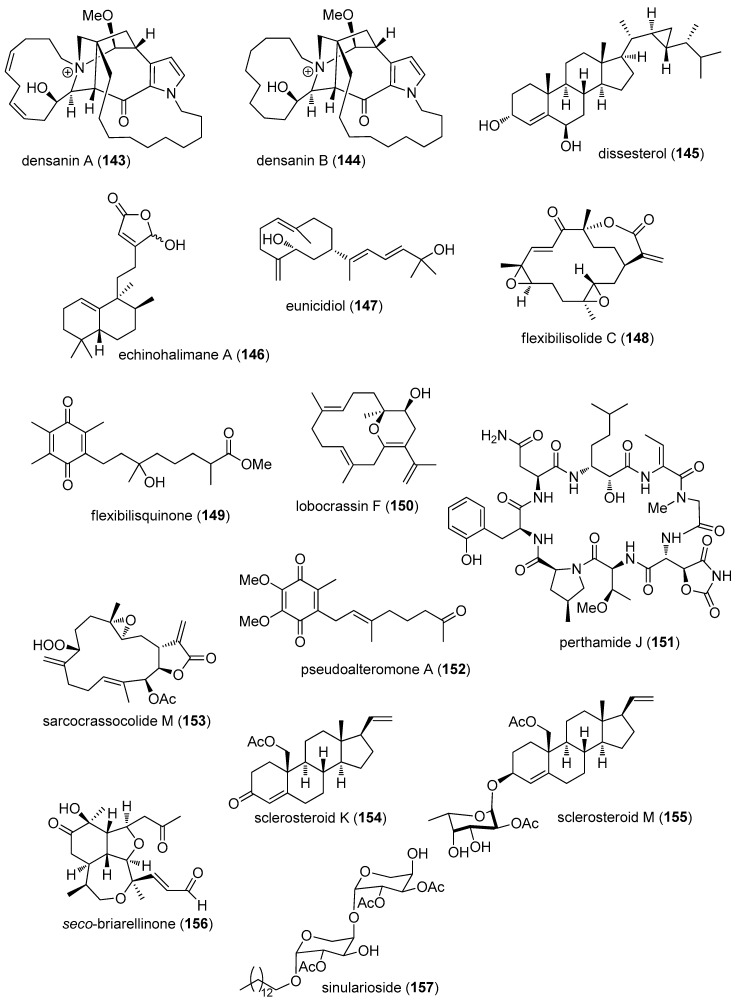

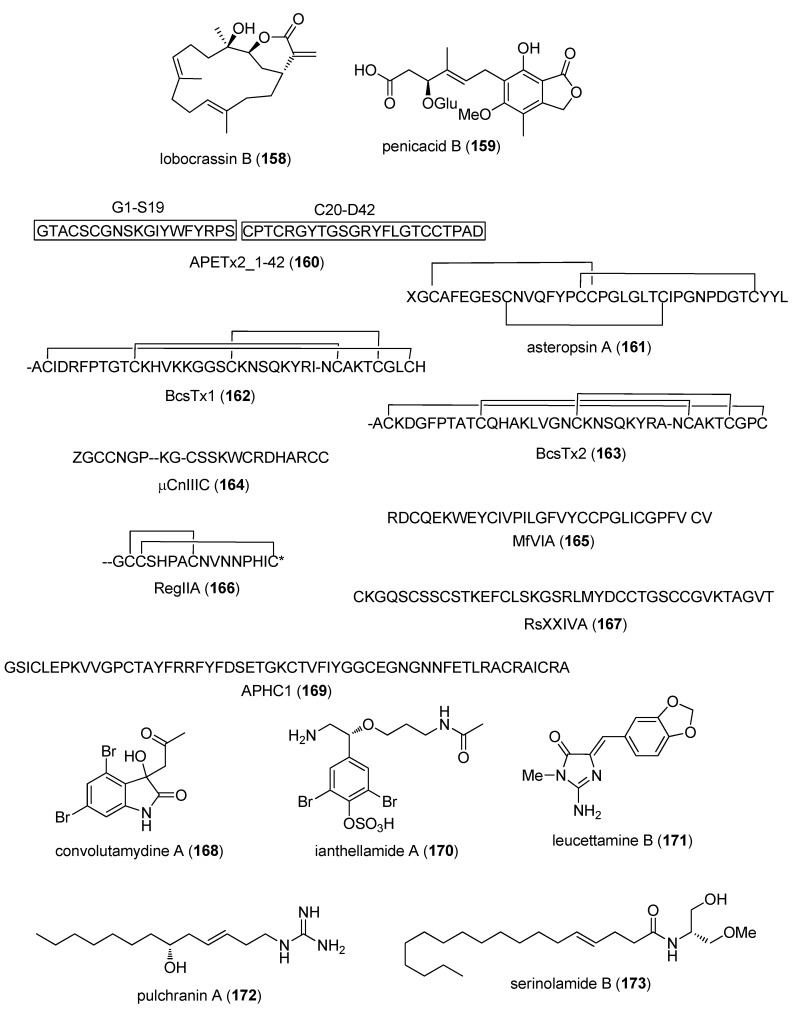

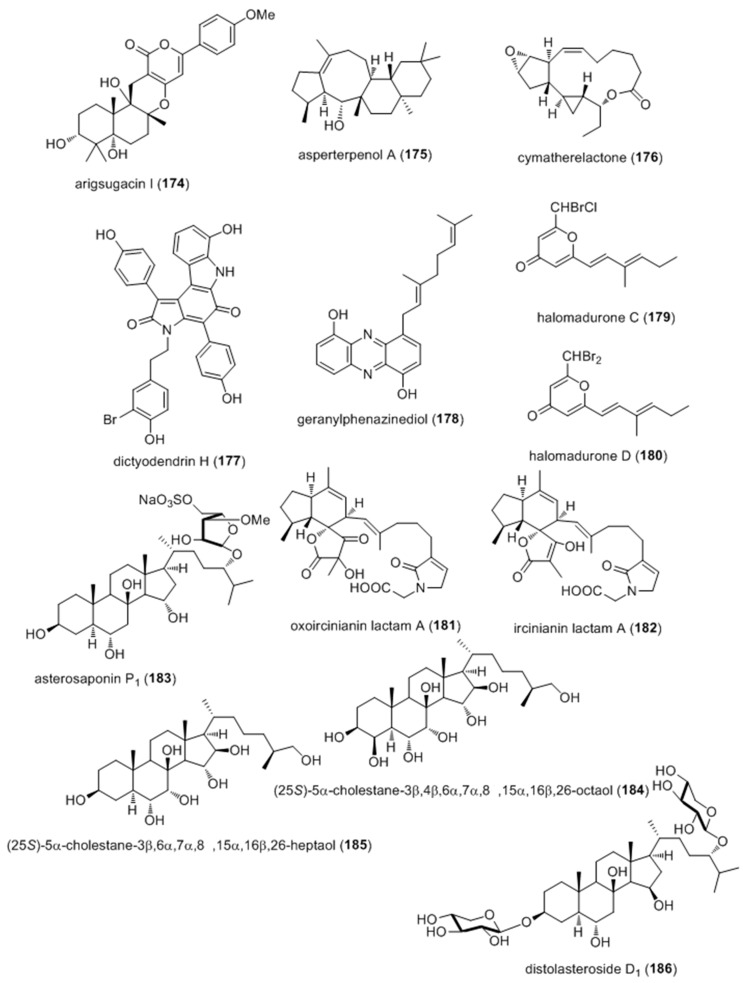

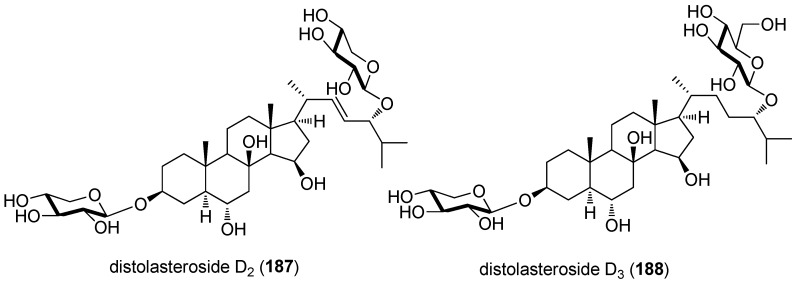
Marine pharmacology in 2012–2013: marine compounds with antidiabetic and anti-inflammatory activity; and affecting the immune and nervous system.

**Figure 3:**
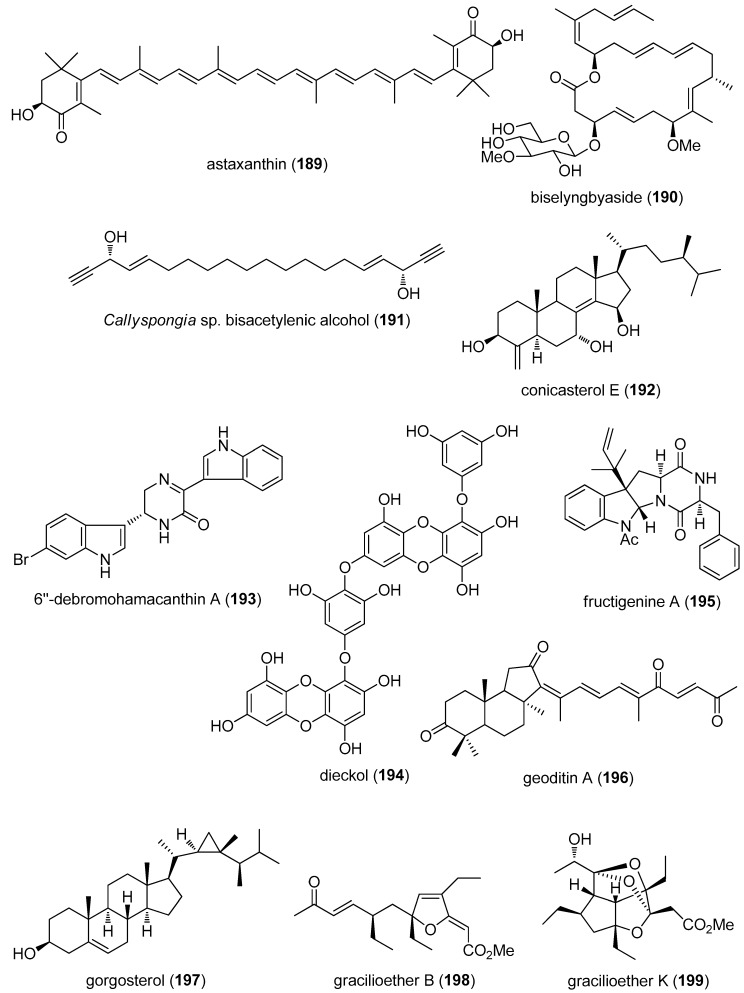

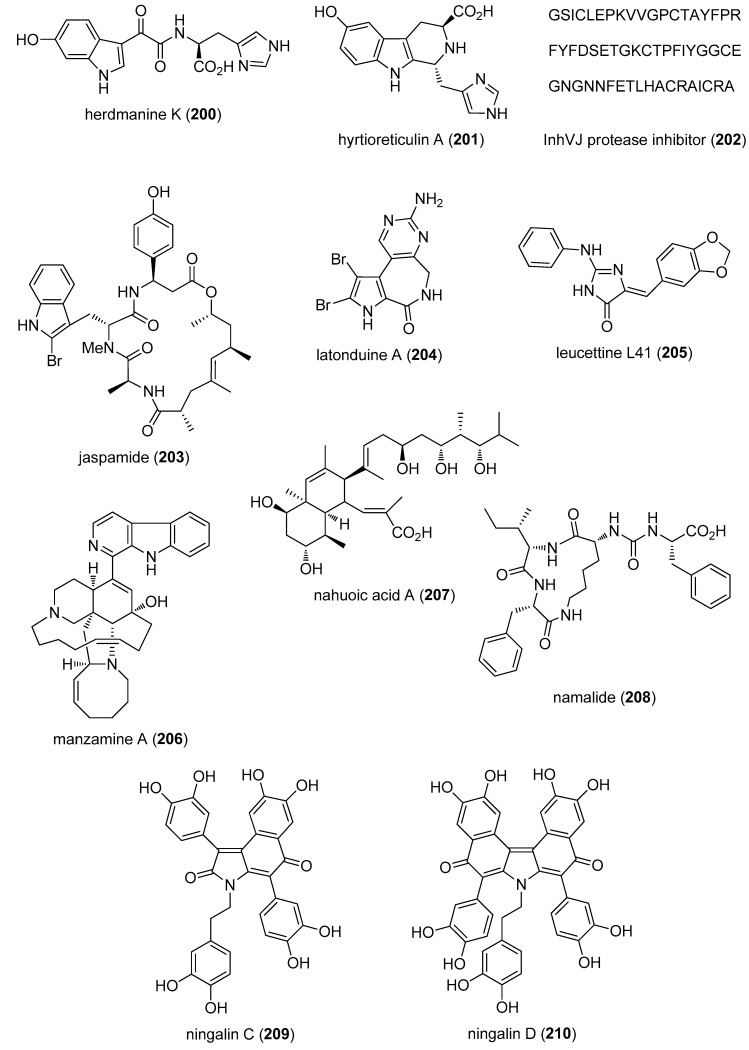

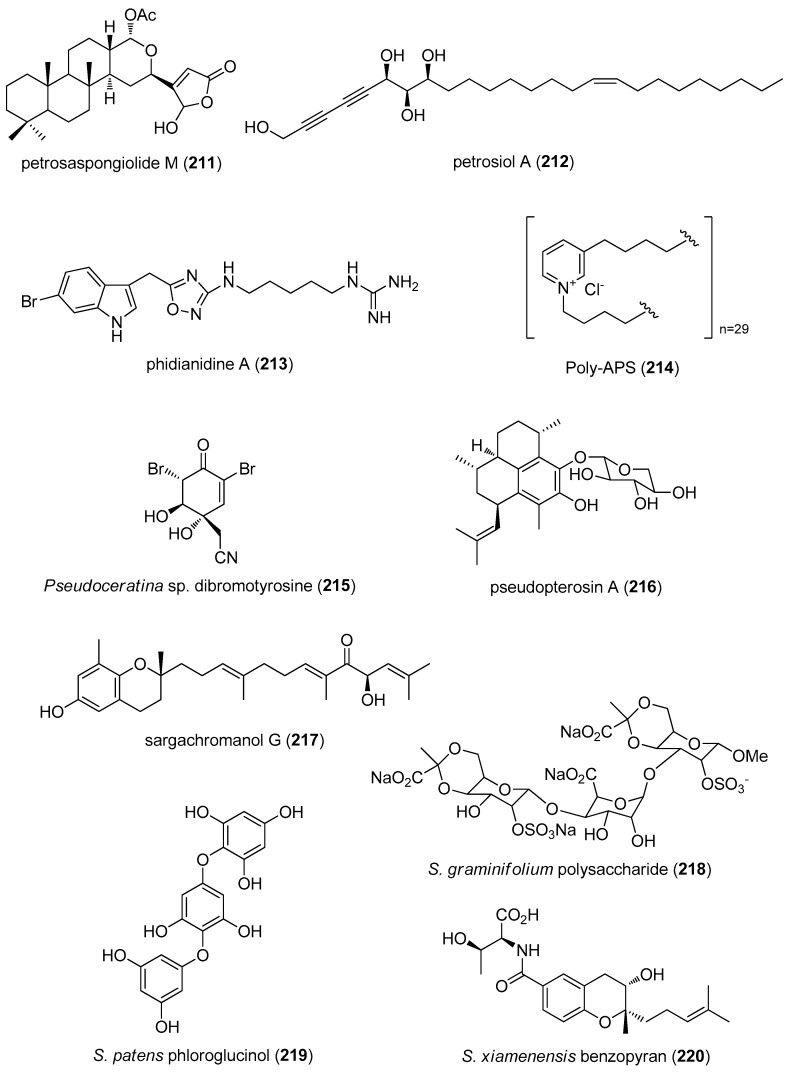

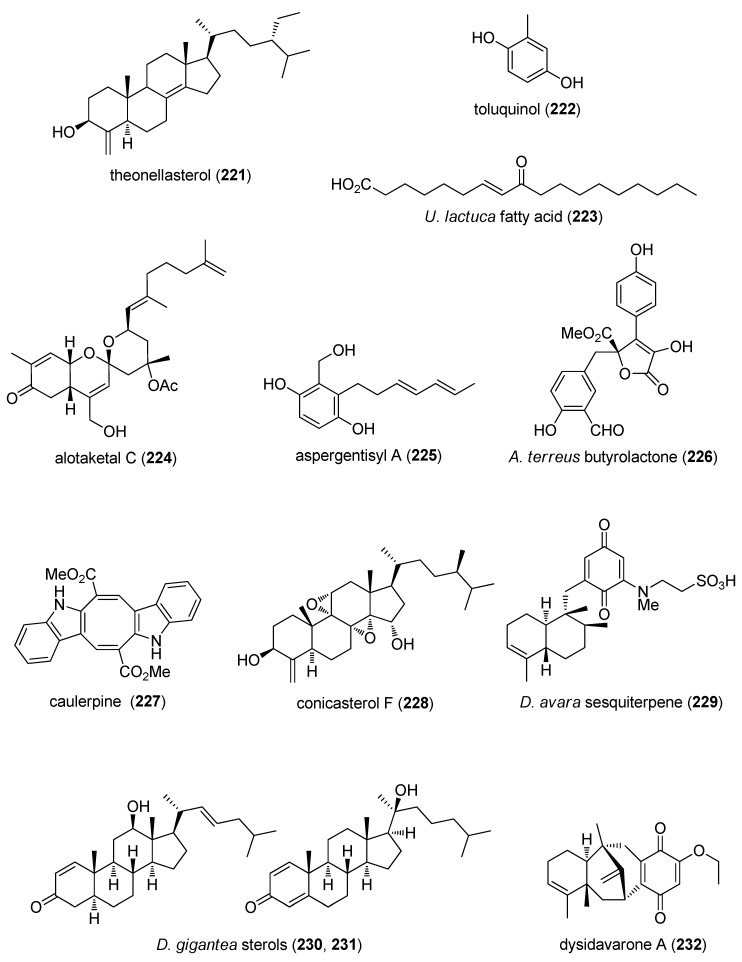

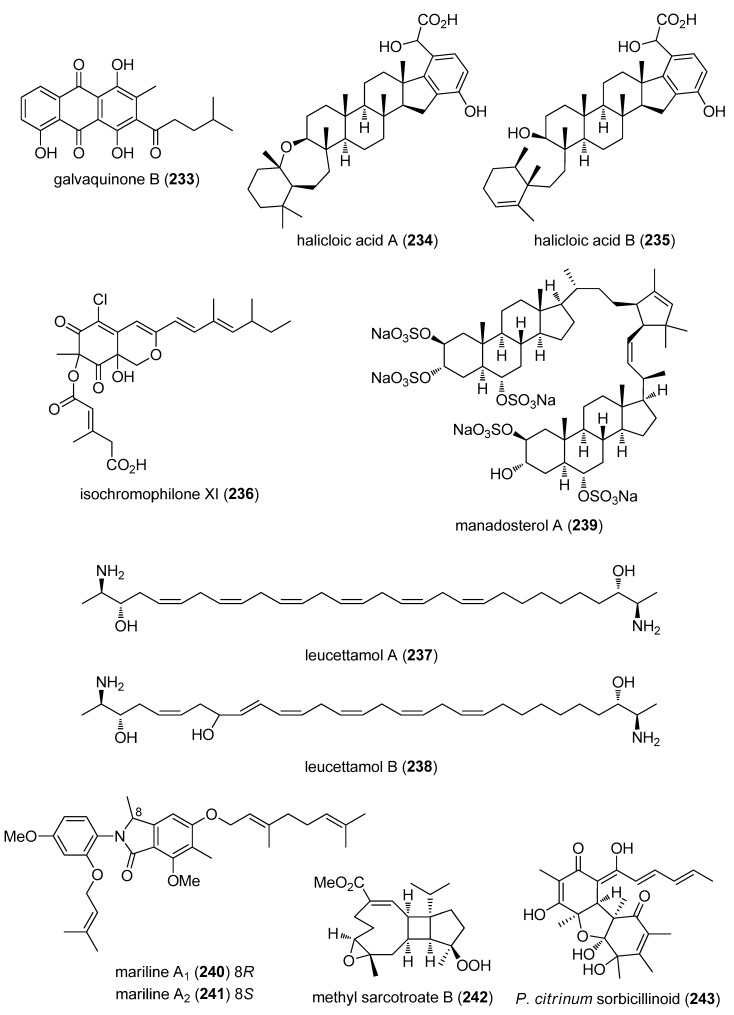

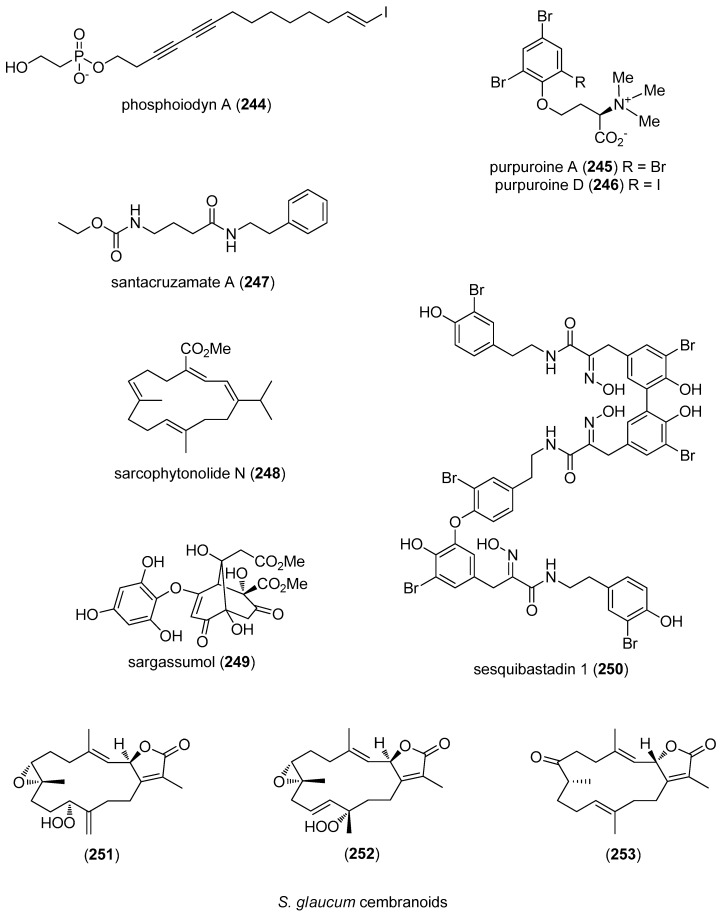

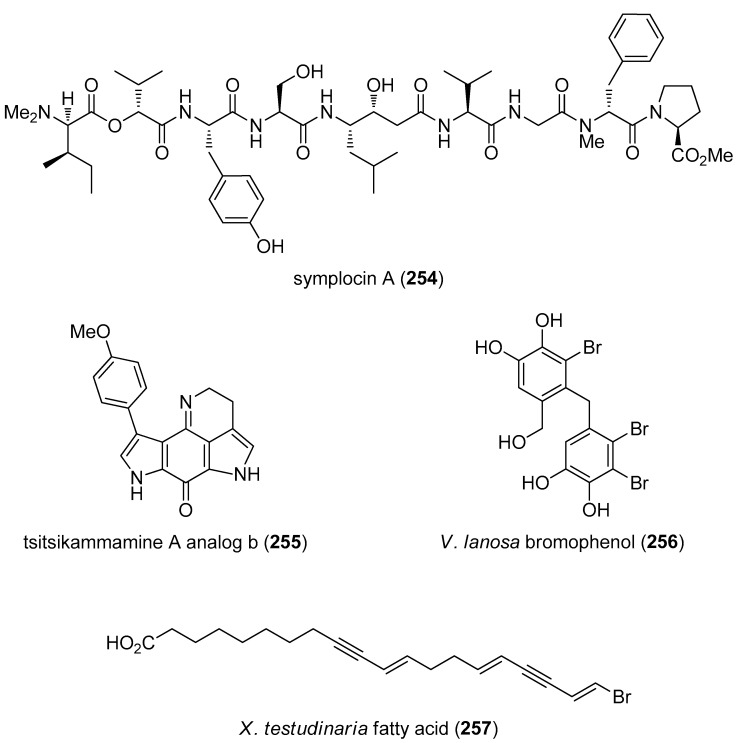
Marine pharmacology in 2012–2013: marine compounds with miscellaneous mechanisms of action.

The aim of the present review is to consolidate *preclinical* marine pharmacology in 2012–2013, with a format similar to the previous 8 reviews of this series, which cover the period 1998–2011 [1,2,3,4,5,6,7,8]. The peer-reviewed articles were retrieved from searches of several databases, including MarinLit, PubMed, Chemical Abstracts^®^, ISI Web of Knowledge and Google Scholar. The review only includes bioactivity and/or pharmacology of structurally characterized marine chemicals, which we have classified using a modification of Schmitz’s chemical classification [9] into six major chemical classes; namely, polyketides, terpenes, peptides, alkaloids, shikimates, and sugars. The preclinical antibacterial, antifungal, antiprotozoal, antituberculosis, antiviral and anthelmintic pharmacology of marine chemicals is reported in Table 1, with the structures shown in Figure 1. Marine compounds that affect the immune and nervous systems, as well as those with antidiabetic and anti-inflammatory effects, are exhibited in Table 2, with their structures presented in Figure 2. Finally, marine compounds that affected a variety of cellular and molecular targets are noted in Table 3, and their structures presented in Figure 3.

A number of publications during 2012–2013 reported extracts or structurally uncharacterized marine compounds, with novel and interesting *preclinical* and/or *clinical* pharmacology: in vitro *antimalarial* activity in crude extracts from Fiji marine organisms using a semi-automated RNA fluorescence-based high-content live cell-imaging assay [10]; the first report of in vitro liver stage *antiplasmodial* activity and dual stage inhibitory potential of British seaweeds [11]; *anti-hepatitis C virus* activity affecting the viral helicase NS3 and replication, in crude extracts from the marine feather star *Alloeocomatella polycladia* [12]; *anti-herpes simplex* virus HSV-1 and HSV-2 activity in a purified sulfoglycolipid fraction from the Brazilian marine alga *Osmundaria obtusiloba* [13]; in vivo *anti-inflammatory* activity of a heterofucan from the Brazilian seaweed *Dictyota menstrualis* that inhibited leukocyte migration to sites of tissue injury by binding to the cell membrane [14]; in vivo *antinociceptive* and *anti-inflammatory* activity in a crude methanolic extract of the red alga *Bryothamnion triquetrum* [15]; in vivo *anti-inflammatory* activity in a sulfate polysaccharide fraction from the red alga *Gracilaria caudata* resulting in significant inhibition of neutrophil migration and cytokine release [16]; in vitro *anti-inflammatory* effect of a hexane-soluble fraction of the brown alga *Laminaria japonica* that inhibited nitric oxide, prostaglandin E_2_, interleukin (IL)-1β and IL-6 release from lipopolysaccharide-stimulated macrophages via inactivation of nuclear factor-κB transcription factor [17]; in vivo *anti-inflammatory* of a polysaccharide-rich fraction from the marine red alga *Lithothamnion muelleri* that reduced organ injury and lethality, as well as pro-inflammatory cytokines and chemokines, associated with graft-versus-host disease in mice [18]; in vivo clinical effectiveness in an osteoarthritis trial by PCSO-524^TM^, a nonpolar lipid extract from the New Zealand marine green lipped mussel *Perna canaliculus*, which may offer “potential alternative complementary therapy with no side effects for osteoarthritis patients” [19]; enhanced *antioxidant* activity of chitosan nanoparticles as compared to chitosan on hydrogen peroxide-induced stress injury in mouse macrophages in vitro [20]; induction of concentration-dependent *vasoconstrictive* activity on isolated rat aorta by a tentacle extract from the jellyfish *Cyanea capillata* [21]; significant *antioxidant* effect of a sulfated-polysaccharide fraction of the marine red alga *Gracilaria birdiae* which prevented naproxen-induced gastrointestinal damage in rats by reversing glutathione depletion [22]; in vitro *antioxidant* properties of a polysaccharide from the brown seaweed *Sargassum graminifolium* (Turn.) that was also observed to inhibit calcium oxalate crystallization, a constituent of urinary kidney stones [23]; *antioxidant* activity in organic extracts from 30 species of Hawaiian marine algae, with the carotenoid fucoxanthin identified as the major bioactive antioxidant compound in the brown alga *T. ornata* [24]; screening of *antioxidant activity* in 18 cyanobacteria and 23 microalgae cell extracts identified *Scenedesmus obliquus* strain M2-1, which protected against DNA oxidative damage induced by copper (II)-ascorbic acid [25]; *anxiolytic*-like effect of a salmon phospholipopeptidic complex composed of polyunsaturated fatty acids and bioactive peptides associated with strong free radical scavenging properties [26]; *antinociceptive* activity in extracts of the skin of the Brazilian planehead filefish *Stephanolepis hispidus* with partial activation of opioid receptors in the nervous system [27]; strong in vitro *acetylcholinesterase* inhibition, an enzyme targeted by drugs used to treat Alzheimer’s disease, myasthenia gravis and glaucoma, by an extract from the polar marine sponge *Latrunculia* sp. [28]; *central nervous system* activity of a phlorotannin-rich extract from the edible brown seaweed *Ecklonia cava* targeting gamma-aminobutyric acid type A benzodiazepine receptors [29]; and novel *protease inhibitors* from Norwegian spring spawning herring determined by screening of marine extracts with assays combining fluorescence resonance energy transfer activity and surface plasmon resonance spectroscopy-based binding [30].

## 2. Marine Compounds with Antibacterial, Antifungal, Antiprotozoal, Antituberculosis, Antiviral and Anthelmintic Activities

Table 1 presents 2012–2013 preclinical pharmacological research on the antibacterial, antifungal, antiprotozoal, antituberculosis, antiviral and anthelmintic activities of marine natural products (**1**–**113**) shown in Figure 1.

### 2.1. Antibacterial Activity

During 2012–2013, 31 studies reported *antibacterial* marine natural products (**1**–**50**) isolated from bacteria, fungi, tunicates, sponges, and algae, a global effort that may contribute to the search for novel leads for developing newer drugs to treat drug-resistant bacterial infections.

As shown in Table 1 and Figure 1, three papers reported molecular mechanism of action studies with marine antibacterial compounds. Jang and colleagues reported a potent antianthrax antibiotic, anthracimycin (**1**), derived from a marine actinomycete with significant activity against *Bacillus anthracis*, by a mechanism that “…remains to be fully defined…” but that appears to involve DNA/RNA synthesis inhibition [31]. Keffer and colleagues extended the mechanism of action of *bis*-diarylbutene macrocycle chrysophaentins (**2**,**3**), isolated from the chrysophyte alga *Chrysophaeum taylori*, by determining that they competitively inhibited the biochemical activity of the Gram-positive and Gram-negative cell division protein FtsZ by binding to its GTP-binding site [32]. Sakoulas and colleagues reported the antibacterial activity of merochlorin A (**4**), a meroterpenoid isolated from a marine-derived actinomycete strain CNH189, which demonstrated activity against Gram-positive bacteria including *Clostridium difficile*, but not against Gram-negative bacteria, by a mechanism that appeared to involve “…global inhibition of DNA, RNA, protein, and cell wall synthesis…” [33].

As shown in Table 1 and Figure 1, 46 marine chemicals (**5**–**50**), some of them novel, were reported to exhibit antibacterial activity with MICs < 10 μg/mL or 10 μM against several bacterial strains, although the mechanism of action for these compounds remained undetermined: a novel aflatoxin B_2b_ (**5**), isolated from the fungus *Aspergillus flavus*; 092008, isolated from the root of the mangrove *H. tiliaceus* from Hainan, China [34]; a new alkaloid ageloxime B (**6**), isolated from the South China Sea marine sponge *Agelas mauritiana* [35]; several known yet bioactive compounds namely altersolanol C (**7**), macrosporin (**8**) and alterporriol C (**9**) isolated from a soft-coral derived from South China Sea fungus *Alternaria* sp. [36]; a novel antimycin A analogue, antimycin B2 (**10**), derived from the actinomycete *Streptomyces lusitanus*, isolated from the mangrove *Avicennia mariana* in Fujian, China [37]; a new bisabolane-type sesquiterpenoid (−)-sydonol (**11**) from a South China Sea sponge-derived fungus *Aspergillus* sp. [38]; three new pyrimidine diterpenes designated axistatins 1 (**12**), 2 (**13**) and 3 (**14**), isolated from the marine sponge *Agelas axifera* collected in the Republic of Palau [39]; two new diterpene-benzoate compounds bromophycoic acid A (**15**) and E (**16**) from a Fijian red alga *Callophycus* sp. [40]; new butenolide cadiolides C–F (**17**–**20**) from a Korean tunicate *Pseudodistoma antinboja* [41]; novel tris-aromatic furanones cadiolides G-I (**21**–**23**) from the Korean dark red ascidian *Synoicum* sp. [42]; xanthones citreamicins *θ* A and B (**24**,**25**), isolated from the Red Sea marine *Streptomyces caelestis* [43]; two new aromatic polyketides, communols A and F (**26**,**27**), isolated from the marine *Penicillium commune* 518, associated with the gorgonian *Muricella abnormalis* [44]; two dolabellane diterpenes (**28**,**29**), isolated from the Greek brown alga *Dilophus spiralis* [45]; a novel enhygrolide A (**30**), isolated from the obligate marine myxobacterium *Enhygromyxa salina* from a mud sample from Prerow, Germany [46]; a new β-carboline alkaloid eudistomin Y_11_ (**31**), isolated from a purple-colored ascidian *Synoicum* sp. [47]; a new capoamycin-type antibiotic fradimycin B (**32**), isolated from the marine *Streptomyces fradiae* strain PTZ0025 [48]; three novel cyclic *bis*-1,3 dialkylpyridiniums (**33**–**35**) from a Korean sponge *Halyclona* sp. [49]; a novel bisindole alkaloid hyrtimomine D (**36**), isolated from an Okinawan marine sponge *Hyrtios* sp. [50]; a new bromotyrosine-derived metabolite, ianthellisformisamine A (**37**), reported from the Australian marine sponge *Suberea ianthelliformis* [51]; a new thiazolyl peptide kocurin (**38**) from the marine-derived bacterium *Kocuria palustris* [52]; the known alkaloid lamellarin O (**39**), isolated from a southern Australian sponge *Ianthella* sp. [53]; three new halogenated sesquiterpenes (**40**–**42**), isolated from the Chinese marine red alga *Laurencia okamurai* [54]; a new spirotetronate antibiotic, lobophorin H (**43**) from a South China Sea-*Streptomyces* sp. 12A35 [55]; a new cyclopeptide marthiapeptide A (**44**), isolated from the South China Sea-derived bacterium *Marinactinospora thermotolerans* [56]; two known napyradiomycin A1 (**45**) and napyradiomycin B3 (**46**) from a Chinese marine-derived *Streptomyces* sp. strain SCSIO [57,58]; two new hydroanthraquinone analogues 4a-*epi*-9α-methoxydihydrodeoxybostrycin (**47**) and 10-deoxy-bostrycin (**48**), isolated from a South China Sea marine-derived fungus *Nigrospora* sp., isolated from an unidentified sea anemone [59]; a novel cyclic peptide ohmyungsamycin A (**49**) from a Korean *Streptomyces* sp. strain SNJ042 [60]; and a novel benzofuran penicifuran A (**50**), obtained from a South China Sea sponge-derived fungus *Penicillium* sp. strain MWZ14-4 [61].

Furthermore, during 2012–2013, several other marine natural products, some of them novel, reported MICs or IC_50_s ranging from 10 to 50 μg/mL, or 10–50 μM, respectively, and thus, because of their lower antibacterial potency, were excluded from Table 1 and Figure 1: guaiazulene-derived terpenoids from a Chinese gorgonian *Anthogorgia* sp. (MIC = 12.7–18 μg/mL) [110]; novel fulvynes antimicrobial polyoxygenated acetylenes from the Mediterranean sponge *Haliclona fulva* (IC_50_ = 12–60 μM) [111]; bioactive polyhydroxylated halicrasterols (MIC = 4–32 μg/mL) from the Chinese marine sponge *Haliclona crassiloba* [112]; hunanamycin A, an antibiotic (MIC = 12.4 μM), isolated from the Bahamanian marine-derived *Bacillus hunanensis* [113]; three new dimeric bromopyrrole alkaloids, nagelamides X–Z (MIC = 8–32 μg/mL) from an Okinawan marine sponge *Agelas* sp. [69]; a new anthraquinone-citrin derivative (MIC = 16 μg/mL), isolated from the sea fan-derived fungus *Penicillium citrinum* PSU-F51 [114]; and a new chlorinated benzophenone derivative, (±)-pestalachloride C (MIC = 5–20 μM) from a South China Sea soft coral-derived fungus *Pestalotiopsis* sp. [115]. Finally, during 2012–2013, the novel marine lipopeptides, peptidolipins B–F (MIC = 64 μg/mL), were isolated from an ascidian-derived Gram positive *Nocardia* sp. bacterium [116].

### 2.2. Antifungal Activity

Eleven studies during 2012–2013 reported on the *antifungal* activity of several novel marine natural products (**6**,**36**,**51**–**60**), isolated from marine fungi, sponges, sea cucumbers and algae, a slight decrease from our last review [7], and previous reviews of this series.

As shown in Table 1 and Figure 1, two reports described antifungal marine chemicals with novel mechanisms of action. Rubiolo and colleagues investigated the guanidine antifungal alkaloid crambescidin-816 (**51**), previously isolated from the Mediterranean sponge *Crambe crambe* [62]. Detailed cell cycle studies in the yeast *Saccharomyces cerevisiae* demonstrated that this compound induced G2/M cell cycle arrest followed by apoptosis and mitochondrial disfunction, suggesting that although cytotoxic crambescidin-816 “….could serve as a lead compound to fight fungal infections”. Yibmantasiri and colleagues investigated the molecular basis for the fungicidal action of the triterpene glycoside neothyonidioside (**52**) isolated from the sea cucumber *Australostichopus mollis* [63], demonstrating that neothyonidioside binds directly to fungal ergosterol affecting membrane curvature and fusion capability essential for membrane recycling and lysosomal degradation.

Furthermore, as shown in Table 1 and Figure 1, several marine natural products showed significant antifungal activity (i.e., MICs that were either less than 10 μg/mL, 10 μM, or 10 μg/disk), although no mechanism of action studies were reported in the published articles: a novel alkaloid ageloxime B (**6**), isolated from the South China Sea sponge *Agelas mauritiana* [35]; a novel tetramic acid glycoside, aurantoside K (**53**), isolated from a Fijian marine sponge *Melophlus* sp. [64]; a new prenylated *para*-xylene caulerprenylol A (**54**), isolated from the green alga *Caulerpa racemosa* collected in the Zhanjiang coastline, China [65]; a new 4-hydroxy-2-pyridone alkaloid didymellamide A (**55**), isolated from the Japanese marine-derived fungus *S. cucurbitacearum* [66]; a new polyketide hippolachnin A (**56**), reported from the South China Sea sponge *Hippospongia lachne* [67]; novel triterpene glycosides holotoxin F and G (**57**,**58**), isolated from the sea cucumber *Apostichopus japonicus* Selenka, “a traditional tonic with high economic value” in China [68]; a novel bisindole alkaloid hyrtimomine D and E (**36**,**59**), isolated from an Okinawan marine sponge *Hyrtios* sp. [50]; a novel dimeric alkaloid nagelamide Z (**60**), isolated from a Japanese sponge *Agelas* sp. [69]; and a new linear polyketide woodylide A (**61**), isolated from the South China Sea sponge *Plakortis simplex* [70]. Ongoing mechanism of action studies with these potent marine compounds will be required to characterize their molecular pharmacology.

Finally, several novel structurally-characterized marine molecules demonstrated MICs or IC_50_s greater than 10 μg/mL, 10 μM, or 10 μg/disk, and therefore, because of the reported weaker antifungal activity, were excluded from Table 1 and Figure 1: three triterpene glycosides, cucumariosides A_1_, A_6_ and A_15_ (MIC = 20 μg/mL), isolated from the Pacific Sea cucumber *Eupentacta fraudatrix* [117]; a tetranorditerpenoid derivative isolated from *Aspergillus wentii* EN-48 (MIC = 16 μg/mL), a fungus isolated from an unidentified marine brown algae [118]; and bromophenol-aconitic acid adduct, symphyocladin G, isolated from the marine red alga *Symphyocladia latiuscula* (MIC = 10 μg/mL) [119]. These novel marine compounds may contribute to ongoing research for clinically useful antifungal agents.

### 2.3. Antiprotozoal and Antituberculosis Activity

As shown in Table 1, during 2012–2013 twenty five studies contributed to novel findings on *antiprotozoal* (*antimalarial*, *antileishmanial and antitrypanosomal*) and *antituberculosis* pharmacology of structurally characterized marine natural products (**62**–**92**), a decrease from previous 1998–2011 reviews [1,2,3,4,5,6,7,8].

Malaria, which is caused by protozoa of the genus *Plasmodium* (*P. falciparum*, *P. ovale*, *P. vivax* and *P. malariae*), affects millions of people worldwide. Contributing to the global search for novel antimalarial drugs, and as presented in Table 1, seventeen novel marine molecules (**62**–**78**), isolated from bacteria, ascidians, fungi, sponges, and tunicates, were shown during 2012–2013 to possess *antimalarial activity*, although mechanism of action studies were not reported for these compounds.

As shown in Table 1 and Figure 1, potent (IC_50_ < 2 μM) to moderate (IC_50_ > 2–10 μM) *antimalarial* activity was reported for several marine natural products (**62**–**78**), isolated from ascidians, sponges, bacteria and fungi. Mani and colleagues reported antiplasmodial activity in the bromotyrosine derivative araplysillin I (**62**) from the South Pacific Solomon Islands sponge *Suberea ianthelliformis* [71]. Lam and colleagues extended the pharmacology of the New Zealand ascidian dioxothiazino-quinoline-quinone metabolite ascidiathiazone A (**63**) by demonstrating it to be a moderate growth inhibitor of chloroquine and a pyrimethamine resistant *P. falciparum* K1 strain, and noting that changing the quinolone-based structure to incorporate benzofuran or benzothiophene moieties yielded particularly potent antimalarials [72]. Farokhi and colleagues characterized new glycosphingolipids axidjiferoside A–C (**64**–**66**) from the Senegal marine sponge *Axinyssa djiferi* with potent antimalarial activity against chloroquine-resistant FcB1/Colombia *P. falciparum* strain [73]. Beau and colleagues reported that epigenetic tailoring of the marine fungus *Leucostoma persoonii* enhanced production of the known polyketide cytosporone E (**67**), which inhibited *P. falciparum* with significant selectivity [74]. Calcul and colleagues reported a massive screening of Chinese mangrove endophytic fungi and discovered several new compounds, including a novel dimeric tetrahydroxanthone polyketide dicerandrol D (**68**), which was potent against “a robust and validated” drug-sensitive *P. falciparum* strain 3D7 [75]. Ilias and colleagues reported a novel pentacyclic ingamine alkaloid dihydroingenamine D (**69**), isolated from a sponge *Petrosid* Ng5 sp.5, which showed strong antiplasmodial activity against *P. falciparum* D6 and W2 strains [76]. Mudianta and colleagues reported that the novel alkaloid 19-hydroxypsammaplysin E (**70**) from the Indonesian marine sponge *Aplysinella strongylata* had notable antimalarial activity against the *P. falciparum* chloroquine-sensitive 3D7 strain [77]. Sirirak and colleagues reported a new trisoxazole macrolide kabiramide L (**71**) from the Thai marine sponge *Pachatrissa nux* that had moderate activity against a *P. falciparum* K1 multidrug-resistant strain [78]. Bharate and colleagues extended the pharmacology of the known meridianin C and G alkaloids (**72**,**73**), originally isolated from the marine tunicate *Aplidium meridianum*, by reporting that they inhibited both chloroquine-resistant D6 and sensitive W2 clones of *P. falciparum* [79]. Liew and colleagues identified orthidine F (**74**), a metabolite from the New Zealand ascidian *Aplidium orthium* of low toxicity and a moderate growth inhibitor of *P. falciparum* K1 strain dual drug-resistant strain [80]. Lin and colleagues isolated a new polyketide endoperoxide plakortide U (**75**) from the Fijian sponge *Plakinastrella mamillaris* with potent antimalarial activity against chloroquine-resistant *P. falciparum* FcM29 strain [81]. Davis and colleagues isolated several novel thiazine alkaloids from the Australian marine sponge *Plakortis lita*, one of which thiaplakortone A (**76**), showed potent activity against the human malaria parasite *Plasmodium falciparum* strains 3D7 and Dd2 with low cytotoxicity [82]. Davis and colleagues reported a novel bispyrroloiminoquinone alkaloid tsitikammamine C (**77**) from an Australian sponge *Zyzzya* sp. that displayed potent activity against *P. falciparum* chloroquine-sensitive 3D7 and -resistant dd2 strains [83]. Supong and colleagues reported a novel *C*-glycosylated benz[*a*]anthraquinone derivative, urdamycinone E (**78**) isolated from a marine *Streptomyces* sp. BCC45596 that potently inhibited *P. falciparum* K1 strain [84].

As shown in Table 1 and Figure 1, nine marine compounds (**79**–**86**) isolated from bacteria, ascidians, sponges, soft corals and algae were reported to possess bioactivity towards so-called neglected protozoal diseases, namely leishmaniasis, caused by the genus *Leishmania* (*L.*), amebiasis, trichomoniasis, and both African sleeping sickness (caused by *Trypanosoma* (*T.*) *brucei rhodesiense* and *T. brucei gambiense*) and American sleeping sickness or Chagas disease (caused by *T. cruzi*).

As shown in Table 1, three reports described four *antitrypanosomal* marine chemicals (**79**–**82**) as well as their mechanisms of action. Sanchez and colleagues examined the mode of action of almiramides (**79**,**80**), originally isolated from the cyanobacterium *Lyngbya majuscula*, and demonstrated for the first time that these compounds inhibited *T. brucei* by disrupting the parasite’s glycosomal function by targeting two membrane proteins, and were thus considered “encouraging candidates for further lead development” [85]. Abdelmohsen and colleagues reported that the dibenzodiazepine alkaloid diazepinomicin (**81**) isolated from a strain of *Micromonospora* sp. RV115 associated with the Croatian marine sponge *Aplysina aerophoba* showed activity against *T. brucei* trypmastigote forms and inhibited the parasite protease rhodesain [86]. Desoti and colleagues extended the pharmacology of (−)-elatol (**82**), a sesquiterpene isolated from the Brazilian red alga *Laurencia dendroidea* shown to affect trypomastigotes of *T. cruzi*, demonstrating that it induced initial depolarization of the parasite’s mitochondrial membrane, followed by an increase in superoxide generation, as well as loss of cell membrane and DNA integrity [87]. 

As shown in Table 1 and Figure 1, five marine natural products (**63**,**83**–**86**) were characterized to exhibit *antileishmanial* and *antiprotozoal* activity, although the mechanism of action remained undetermined. Lam and colleagues reported that the known dioxothiazino-quinoline-quinone metabolite ascidiathiazone A (**63**), isolated from a New Zealand ascidian, moderately inhibited the growth of *T. brucei rhodesiense*, but was ineffective against *T. cruzi* and *L. donovani* [72]. Balunas and colleagues isolated the polyketide coibacin A (**83**) from a Panamanian marine cyanobacterium *Oscillatoria* sp., and observed potent activity against *L. donovani* axenic amastigotes [88]. Ishigami and colleagues isolated a new xenicane diterpenoid cristaxenicin A (**84**) from the deep-sea gorgonian *Acanthoprimnoa cristata*, which showed potent activity against *L. amazonensis* and *T. congolense* [89]. Chianese and colleagues completed structure-activity relationship studies with several natural and semisynthetic manadoperoxide B analogues (**85**,**86**), isolated from the Indonesian sponge *Plakortis* sfr. *lita*, and determined that both were highly active towards the parasite *T. brucei rhodesiense*, highlighting the 1,2-dioxane ring to be a key pharmacophore [90]. 

Because of the surge in drug-resistant strains of the intracellular pathogen *Mycobacterium tuberculosis* (*Mtb*), there is a global need for the development of novel drugs with novel mechanisms of action. As shown in Table 1 and Figure 1, seven novel marine natural products (**78**,**87**–**92**), isolated from bacteria, sponges and fungi, contributed to the ongoing global search for novel *antituberculosis* agents. Although these marine natural products were characterized to exhibit *antituberculosis* activity, unfortunately the mechanism of action of these compounds remained undetermined.

Huang and colleagues reported a novel sesterterpenoid asperterpenoid A (**87**) from a mangrove endophytic fungus *Aspergillus* sp. that demonstrated strong inhibitory activity against *M. tuberculosis* protein tyrosine phosphatase B, an enzyme that is “…considered a promissory target for pulmonary tuberculosis cure” [91]. Song and colleagues isolated a new dimeric diketopiperazine, brevianamide S (**88**), from *Aspergillus versicolor* collected in the Bohai Sea, China, which demonstrated selective antibacterial activity against Bacille Calmette-Guérin (BCG), “suggestive of a new mechanism of action that could inform the development of next generation antitubercular drugs…if translated to *M. tuberculosis*…” [92]. Chen and colleagues reported a new spirotetronate, lobophorin G (**89**), from a marine-derived *Streptomyces* sp. MS100061 which exhibited strong anti-*M. bovis* BCG activity, providing relevant pharmacological information as this screen is thought to “serve as a useful screening surrogate for *M. tuberculosis*” [93]. Yamano and colleagues discovered a new cyclic depsipeptide neamphamide B (**90**) in a Japanese marine sponge *Neamphius* sp., which showed activity against *M. bovis* BCG in “both actively growing and dormant states” [94]. Avilés and colleagues isolated two new tricyclic diterpenes (**91**,**92**) from the Bahamian marine sponge *Svenzea flava* that displayed moderate antimycobacterial activity against *M. tuberculosis* H37Rv, the data suggesting that “the isoneoamphilectane backbone” may be “responsible for the observed activity” [95]. In addition to the antimalarial activity described earlier, Supong and colleagues reported that the novel *C*-glycosylated benz[*a*]anthraquinone derivative, urdamycinone E (**78**), inhibited *M. tuberculosis* strain H37Rv [84].

### 2.4. Antiviral Activity

As shown in Table 1 and Figure 1, thirteen reports were published during 2012–2013 on the *antiviral* pharmacology of marine natural products (**93**–**102**) against hepatitis C, human immunodeficiency virus type-1 (HIV-1), influenza virus, human rhinovirus (HRV) and herpes simplex virus (HSV). 

As shown in Table 1, only six reports described antiviral marine chemicals and their mechanisms of action. Da Rosa Guimarães and colleagues extended the pharmacology of the known steroids halistanol sulfate (**93**) and halistanol sulfate C (**94**), isolated from the Brazilian marine sponge *Petromica citrina*, by demonstrating that the compounds inhibited attachment and penetration of the “early events of HSV-1 infection” [96]. Ellithey and colleagues investigated several known metabolites (**95**–**97**) from the Red Sea soft coral *Litophyton arboreum* and demonstrated selective inhibition of the HIV-1 protease by a mechanism that “confirms the contribution of the hydrophobicity of inhibitors of HIV protease” [97]. Salam and colleagues reported a novel pharmacological activity for the sesterterpene manoalide (**98**), which was observed to affect the hepatitis C virus NS3 helicase by inhibiting RNA binding and ATPase activity [98]. Park and colleagues reported that two polybromocatechol compounds (**99**,**100**), isolated from the red alga *Neorhodomela aculeate*, inhibited infection and cytopathic effects on a HeLa cell line by HRV2 and HRV3, causal agents of viral respiratory infections and common colds [99]. Ma and colleagues determined that the novel phenylspirodrimane stachybotrin D (**101**), isolated from the fungus *Stachybotrys chartarum* MXH-X73 derived from the Chinese marine sponge *Xestospongia testudinaria*, inhibited HIV-1 replication of wild-type and five non-nucleoside reverse transcriptase inhibitor (NNRTI)-resistant HIV-1 strains by inhibiting the reverse transcriptase, and thus “provides a new class of chemotype for the search of NNRT inhibitors” [100]. Jiao and colleagues reported that streptoseolactone (**102**), derived from the actinomycete *Streptomyces seoulensis* strain isolated from the shrimp *Penasus orientalis*, inhibited neuraminidase by a noncompetitive mechanism, a finding “of value in terms of drug discovery for the treatment of influenza” [101].

As shown in Table 1 and Figure 1, several marine natural products (**103**–**111**) were characterized to exhibit antiviral activity, although the mechanism of action of these compounds remained undetermined. He and colleagues isolated a novel cyclic tetrapeptide asperterrestide A (**103**) from the marine-derived fungus *Aspergillus terreus* SCSGAF0162, which inhibited influenza virus strains H1N1 and H3N2 [102].

Two contributions by Peng and colleagues reported two novel indole alkaloids (**104**,**105**), produced by the mangrove-derived fungus *Cladosporium* sp. PJX-41, that inhibited influenza A virus H1N1 [103], and a new pyronepolyene *C*-glucoside iso-D8646-2-6 (**108**), from a sponge-associated fungus *Epicoccum* sp. JJY40, that also inhibited the influenza virus H1N1 [106]. Hawas and colleagues isolated the novel isorhodoptilometrin-1-methyl ether (**106**) from the Red Sea marine fungus *Aspergillus versicolor*, which exhibited hepatitis virus C NS3/4A protease activity [104]. Zhang and colleagues isolated a novel polyketide massarilactone H (**107**) from the marine-derived fungus *Phoma herbarum* which displayed moderate neuraminidase inhibitory activity [105]. Ahmed and colleagues purified a novel polyhydroxylated sterol (**109**) and a new ceramide (**110**) from the Red Sea soft coral *Sinularia candidula*, which inhibited the H5N1 avian influenza viral strain [107]. Plouguerné and colleagues characterized the antiviral activity of a sulfoquinovosyldiacylglycerol (**111**) from the Brazilian brown seaweed *Sargassum vulgare*, demonstrating that it inhibited both HSV-1 and HSV-2 more potently than acyclovir, a clinically used antiherpetic agent [108].

### 2.5. Anthelmintic Activity

As shown in Table 1, only one report was published during 2012–2013 on the *anthelmintic* pharmacology of marine natural products. Melek and colleagues isolated triterpene glycosides echinosides A and B (**112**,**113**) from the sea cucumbers *Actinopyga echinites* and *Holothuria polii* that displayed “potential in vitro schisotomicidal activity against worms of *Schistosoma mansoni*”, suggesting that these compounds may be “promising lead compounds for the development of new schistosomicidal agents” [109].

## 3. Marine Compounds with Antidiabetic and Anti-Inflammatory Activity, and Affecting the Immune and Nervous System

Table 2 presents the 2012–2013 preclinical pharmacology of marine chemicals (**114**–**188**), which demonstrated either antidiabetic or anti-inflammatory activity, as well as those affecting the immune or nervous system; their structures are depicted in Figure 2.

### 3.1. Antidiabetic Activity

Lee and colleagues reported the pharmacology of octaphlorethol A (**114**), a novel phenolic compound isolated from the marine brown alga *Ishige foliacea*, by showing that octaphlorethol A enhanced glucose uptake in L6 rat myoblast cells by increasing glucose transporter 4 translocation to the plasma membrane and protein kinase B and AMP-activated protein kinase activity [120].

### 3.2. Anti-Inflammatory Activity

As shown in Table 2 and Figure 2, there was a remarkable increase in marine anti-inflammatory pharmacology research during 2012–2013. The molecular mechanism of action of marine natural products (**115**–**134**) was investigated in both in vitro and in vivo preclinical pharmacological studies which were completed using a variety of in vitro models including bone marrow-derived macrophages, human U937 monocytic cells, murine RAW 264.7 macrophages, human epidermoid carcinoma A431 cell line, human polymorphonuclear leukocytes, rat brain microglia, and mouse peritoneal macrophages. 

Chae and colleagues evaluated the anti-inflammatory properties of apo-9′-fucoxanthinone (**115**), isolated from the marine edible brown alga *Sargassum muticum* [121] in unmethylated CpG DNA-stimulated bone marrow-derived macrophages and dendritic cells. Inhibition of interleukin-12 p40, interleukin-6 (IL-6) and tumor necrosis factor-α (TNF-α) production, as well as concomitant attenuation of the mitogen-activated protein kinase pathways, was observed, leading the authors to conclude that apo-9′-fucoxanthinone may have “potential therapeutic use…for inflammatory disease”. In a detailed mechanistic study, Speranza and colleagues investigated the antioxidant marine carotenoid astaxanthin (**116**), showing that it inhibited hydrogen peroxide-stimulated production of pro-inflammatory cytokines IL-1, IL-6 and TNF-α in a human U937 monocytic cell line by selectively restoring physiological levels and function of the tyrosine phosphatase SHP-1, thus proposing that astaxanthin might become a novel agent for the therapy of inflammatory diseases [122]. Johnson and colleagues identified the alkaloids bengamide A and B (**117**,**118**) as potent inhibitors of NFκB and LPS-induced expression of cytokines IL-6, TNF-α and chemokine monocyte chemoattractant protein-1 (MCP-1) release from murine RAW 264.7 macrophages, concluding that these compounds may “serve as therapeutic leads for immune disorders involving inflammation” [123]. Song and colleagues determined that bis-*N*-norgliovictin (**119**) derived from a marine fungus *S3-1-c* inhibited TNF-α, IL-6, interferon-β, and MCP-1 production by LPS-stimulated RAW 264.7 macrophages and affecting Toll-like receptor 4 (TLR-4) signal transduction pathways, as well as LPS-induced septic shock in mice, thus suggesting bis-*N*-norgliovictin might result in a useful therapeutic candidate for “sepsis and other inflammatory diseases” [124]. Investigations by Yang and colleagues with phlorotannin 6,6’-bieckol (**120**), isolated from the marine brown alga *Ecklonia cava*, showed that the compound inhibited expression and release of nitric oxide, prostaglandin E_2_, TNF-α and IL-6 in LPS-stimulated macrophages, with concomitant inhibition of NFκB activation, suggesting that compound **120** is potentially useful for the treatment of inflammatory diseases [125]. Balunas and colleagues determined that the polyketide coibacin B (**121**), isolated from the Panamanian marine cyanobacterium, cf. *Oscillatoria* sp. possessed not only antileishmanial activity, but also significant anti-inflammatory activity, as it significantly decreased LPS-induced nitric oxide, TNF-α and IL-6 release from RAW 264.7 macrophages [88]. Hsu and colleages reported that the soft coral *S. flexibilis*-derived 11-*epi*-sinulariolide acetate (**122**) inhibited cyclooxygenase-2 and interleukin-8 expression in human epidermoid carcinoma A431 cells in vitro by inhibition of Ca^2+^ signaling, suggesting that it might become a lead compound to target “store-operated calcium signaling-dependent inflammatory diseases” [126]. Choi and colleagues demonstrated that the novel honaucin A (**123**) from the Hawaiian cyanobacterium *Leptolyngbya crossbyana*, which inhibited LPS-induced nitric oxide production, and TNF-α, IL-1β, IL-6 and iNOS gene transcription in RAW 264.7 macrophages, had functional groups “critical for anti-inflammatory... activity” [127]. Rat brain microglia, a macrophage type involved in neuroinflammation and neurodegeneration [180] was used by Mayer and colleagues to investigate several known diterpene isocyanide amphilectane metabolites (**124**,**125**) from the Caribbean marine sponge *Hymeniacidon* sp., which potently inhibited thromboxane B_2_ generation from LPS activated rat neonatal microglia in vitro, with concomitant low lactate dehydrogenase release and minimal mitochondrial dehydrogenase inhibition. The authors concluded that the potency of these compounds warranted “further investigation…as lead compounds to modulate…activated microglia in neuroinflammatory disorders” [128]. Ahmed and colleagues extended the pharmacology of largazole (**126**), originally isolated from a marine cyanobacterium *Symploca* sp., by reporting that largazole inhibited class I histone deacetylase 6 in vitro in human rheumatoid arthritis. Furthermore, largazole-enhanced expression of intercellular adhesion molecule-1 and vascular cell adhesion molecule-1 was observed to be mediated by activation of the p38 and Akt signal transduction pathways in synovial fibroblasts [129]. Lee and colleagues reported that the sesquiterpenoid lemnalol (**127**), isolated from the Japanese soft coral *Lemnalia tenuis*, attenuated monosodium urate-induced gouty rat arthritis, by a mechanism that involved inhibition of inducible nitric oxide synthase and cyclooxygenase-2, thus becoming a potential new candidate for “development of a new treatment for gout” [130]. Kim and colleagues reported that the diketopiperazine-type indole alkaloid neoechinulin A (**128**), isolated from an Antarctic marine fungus *Eurotium* sp. SF-5989, inhibited LPS-stimulated RAW264.7 macrophages expression, release of nitric oxide and prostaglandin E_2_, with concomitant inhibition of NFκB activation, and reduced inhibitor NFκB and p38 mitogen-activated protein kinase (MAPK) phosphorylation [131]. In a detailed study, Lee and colleagues investigated penstyrylpyrone (**129**), isolated from a marine-derived fungus *Penicillium* sp. JF-55, and determined that the inhibition of LPS-treated murine peritoneal macrophage production of NO, PGE_2_, TNF-α, IL-1β, was correlated with suppression of iκB-α and NF-κB and concomitant expression of heme oxygenase-1 [132]. Vilasi and colleagues extended the molecular pharmacology of the novel cyclic octapepetide perthamide C (**130**), isolated from the marine sponge *Theonella swinhoei*, by investigating its effect on the proteome of murine macrophages J774.A1 using two-dimensional proteomics, and determining differential effect on several cytosolic and ER-associated proteins, mainly involved in cellular folding processes, thus “shed(ding) more light on the…mechanisms of action” of this natural product [133]. Reina and colleagues reported that *R*-prostaglandins (**131**,**132**) isolated from the Caribbean Colombian soft coral *Plexaura homomalla* inhibited 12-*O*-tetradecanoylphorbol-13-acetate-induced mouse ear inflammation in vivo and decreased human polymorphonuclear leukocytes degranulation, as well as myeloperoxidase and elastase levels in vitro, thus concluding that prostaglandins from “…*P. homomalla* are promising molecules with an interesting anti-inflammatory activity profile” [134]. Huang and colleagues extended the pharmacology of the known compound sinularin (**133**), demonstrating that it modulates nociceptive responses and spinal neuroinflammation by a mechanism that may involve inhibition of leukocyte iNOS and cyclooxygenase-2 (COX-2) and the upregulation of the anti-inflammatory cytokine transforming growth factor-β [135]. Marino and colleagues reported the molecular pharmacology of the novel polyhydroxylated steroid swinhosterol B (**134**) isolated from the Solomon Islands marine sponge *T. swinhoei* [136]. Swinhosterol B was shown to be a highly specific agonist for the human pregnane-X-receptor (PXR), and in transgenic PXR murine monocytes, it attenuated pro-inflammatory cytokine production in vitro, thus supporting “the exploitation of this compound in rodent model(s) of liver inflammation and cholestasis”.

As shown in Table 2, and in contrast to the 20 marine compounds (**115**–**134**) with described anti-inflammatory mechanisms of action, for marine compounds (**135**–**157**), only anti-inflammatory activity, namely IC_50_, was reported, but the molecular mechanism of action remained undetermined: *A. polyacanthus* steroids (**135**,**136**) [137]; barettin (**137**) [138]; briarenolide F (**138**) [139]; diketopiperazine (**139**) [140]; 6-*epi*-cladieunicellin F (**140**) [141]; crassarosteroside A (**141**) [142]; cystodione A (**142**) [143]; densanins A and B (**143**,**144**) [144]; dissesterol (**145**) [145]; echinohalimane A (**146**) [146]; eunicidiol (**147**) [147]; flexibilisolide C (**148**) [148]; flexibilisquinone (**149**) [149]; lobocrassin F (**150**) [150]; perthamide J (**151**) [151]; pseudoalteromone A (**152**) [152]; sarcocrassocolide M (**153**) [153]; sclerosteroids K and M (**154**,**155**) [154]; seco-briarellinone (**156**) [155]; and sinularioside (**157**) [156].

### 3.3. Marine Compounds with Activity on the Immune System

In 2012–2013 preclinical pharmacology of marine compounds that affected the *immune* system showed a decline as previously reported in this series.

Lin and colleagues reported that the cembrane-type diterpenoid lobocrassin B (**158**), isolated from the marine soft coral *Lobophytum crissum*, demonstrated immunomodulatory effects on bone marrow-derived dendritic cells (DC), a cell type known to be an important link between the innate and adaptive immune response [157]. Lobocrassin B was shown to attenuate DC maturation and activation with concomitant inhibition of toll-like receptor-stimulated translocation of NF-κB and TNF-α production, data that suggested that lobocrassin B might have “therapeutic applications in certain immune disfunctions”. Chen and colleagues reported that a novel mycophenolic acid derivative, penicacid B (**159**), isolated from a South China sea fungus *Penicillium* sp. SOF07, inhibited splenocyte lymphocyte proliferation by a mechanism that involved inhibition of inosine 5′-monophosphate dehydrogenase, an essential rate-limiting enzyme in purine metabolic pathway and an “important drug target for immunosuppressive” activity [158]. 

### 3.4. Marine Compounds Affecting the Nervous System

In 2012–2013, the preclinical marine *nervous* system pharmacology with compounds (**160**–**188**), which is consolidated in Table 2 and Figure 2, was focused on sodium and potassium channels, nicotinic acetylcholine receptors, as well as, analgesia, antinociception, and neuroprotection. 

Four marine compounds (**160**–**163**) were shown to bind to sodium (Na^+^) and potassium (K^+^) channels. Jensen and colleagues determined the effect of cyclisation on the stability of the sea anemone peptide APETx2 (**160**). Cyclization with either a six-, seven- or eight-residue linker appeared to be a “promising strategy” to increase protease resistance of APETx2, but it decreased its potency against non-voltage gated, pH-sensitive Na^+^ channel ASIC3 (IC_50_ = 61 nM). Furthermore, truncation at either *N*- and *C*-terminus significantly affected APETx2 binding to ASIC3, demonstrating their critical role in this process [159]. Li and colleagues reported the discovery of a cysteine-crosslinked peptide asteropsin A (**161**), isolated from a Korean marine sponge *Asteropus* sp., that affected neuronal Ca^2+^ influx by a mechanism that involved murine cerebrocortical neurons agonist-induced Na^+^ channel activation, and may thus represent “…a valuable contribution to the cysteine knot peptide-based drug development as a model scaffold” [160]. Orts and colleagues published the biochemical and electrophysiological characterization of two novel sea anemone type 1 potassium toxins, namely Bcs Tx1 (**162**) and Bcs Tx2 (**163**) isolated from the Atlantic sea anemone *Bunodosoma caissarum*, and demonstrated by electrophysiological screening of 12 subtypes of voltage-gated Kv K^+^ channels, that BcsTx1 showed highest affinity for rKv1.2 (IC_50_ = 0.03 ± 0.006 nM) while Bcs Tx2 potently inhibited rKv1.6 (IC_50_ = 7.76 ± 1.90 nM) [161]. 

Four studies extended the pharmacology of conopeptides (**164**–**167**). Favreau and colleagues reported that a novel μ-conopeptide CnIIIIC (**164**) isolated from the venom of the marine snail *C. consors* strongly decreased mouse hemidiaphragm contraction by a mechanism that involved potently blocking muscle Na_v_1.4 (IC_50_ = 1.3 nM) and rat brain Na_v_1.2 (IC_50_ < 1 μM) voltage-gated Na^+^ channels in a “virtually irreversible” manner, which will probably result in potential development of **164** “…as a myorelaxing drug candidate” [162]. Vetter and colleagues reported the isolation and characterization of a novel hydrophobic 32-residue μO-conotoxin MfVIA (**165**), isolated from the venom of marine snail *C. magnificus*, and by using a variety of electrophysiological techniques demonstrated that it preferentially inhibited Nav1.8 (IC_50_ = 96 nM) and Nav1.4 (IC_50_ < 81 nM) voltage-gated Na^+^ channels, leading the authors to propose it as a “drug lead for development of improved analgesic molecules… to improve pain management” [163]. Franco and colleagues isolated an α4/7-conotoxin RegIIA (**166**) from the venom of the marine cone snail *C. regius*, and demonstrated that it potently inhibited α3β4 neuronal nicotinic acetylcholine receptors (IC_50_ = 33 nM) by a mechanism that will require continuous investigation to determine “the precise binding mode of this peptide” [164]. Bernáldez and colleagues described the isolation and biochemical characterization of the first *Conus regularis* conotoxin designated RsXXIVA (**167**) with an eight-cysteine framework, which “diverges from other known conotoxins” and that inhibited Ca_v_2.2 channels (IC_50_ = 2.8 μM) in rat superior cervical ganglion neurons, and also displayed both analgesic and anti-nociceptive activity in the hot-plate and formalin murine in vivo assays, which may contribute to the “design of analgesic peptides” [165].

Two studies reported marine compounds (**168**,**169**) that contributed to nociceptive pharmacology. Figuereido and colleagues extended the pharmacology of convolutamydine A (**168**), isolated from the Floridian marine bryozoan *Amantia convoluta*, demonstrating that it caused peripheral anti-nociceptive and anti-inflammatory effects in several acute pain models, an effect probably mediated by the cholinergic, opioid and nitric oxide systems and “comparable to morphine’s effects” [166]. Andreev and colleagues contributed an extensive in vitro and in vivo pharmacological study of two polypeptides APHC1 and PAHC3 (**169**), isolated from the sea anemone *Heteractis crispa*, shown to have significant anti-nociceptive and analgesic activity in a number of in vivo murine models with associated hypothermia. Furthermore, the two compounds were proposed as a new class of vanilloid 1 receptors modulators based on detailed in vitro biochemical studies [167]. 

Neuroprotective activity of marine compounds (**170**,**171**) was reported in two studies. Feng and colleagues observed that the novel octopamine derivative ianthellamide A (**170**), isolated from the Australian marine sponge *Ianthella quadrangulate*, increased endogenous kynurenic acid in rat brain, as well as selectively inhibited the kynurenine 3-hydroxylase in vitro, thus revealing that modulation of the kynurenine pathway of tryptophan metabolism by this compound suggested “potential as a neuroprotective agent” [168]. Burgy and colleagues completed an extensive pharmacological study on the selectivity, co-crystal structures and neuroprotective properties of the leucettines, analogues of the marine sponge alkaloid leucettamine B (**171**), originally isolated from the calcareous sponge *Leucetta microraphis.* An optimized product, leucettine L41, with multi-target selectivity that resulted in neuroprotective effects was proposed for “further optimization as potential therapeutics against neurodegenerative diseases such as Alzheimer’s disease” [169]. 

As shown in Table 2, additional marine compounds (**172**–**174**) were shown to modulate other molecular targets, i.e., TRPV1 and cannabinoid receptors, and the acetylcholinesterase enzyme. Guzii and colleagues reported that a novel guanidine-containing compound pulchranin A (**172**), isolated from the marine sponge *Monanchora pulchra* inhibited TRPV1 receptor, an ionic channel involved in the regulation of pain and body temperature. Pulchranin A, “the first marine non-peptide inhibitor of TRPV1 channels”, led to a decrease of Ca^2+^ response in a CHO cell line expressing the rat TRPV1 channel by a mechanism the authors propose may result from “direct action on the channel pore” [170]. Montaser and colleagues reported a new fatty acid amide, serinolamide B (**173**), isolated from the Guam cyanobacterium *Lyngbya majuscula* that bound with higher selectivity to cannabinoid receptor CB2 and inhibited forskolin-stimulated cAMP accumulation in Chinese hamster ovary cells expressing the CB1 and CB2 receptors, a finding that “introduces a new structural lead to the cannabimimetic” field of research [171]. Huang and colleagues reported the isolation of a new α-pyrone meroterpene arigsugacin I (**174**), isolated from an endophytic fungus *Penicillium* sp. Sk5GW1L [172] that was observed to potently inhibit acetylcholinesterase, thus contributing to the “best-established treatment target for the design of anti-Alzheimer’s drugs”.

In contrast to the 15 marine compounds (**160**–**174**) affecting the nervous system with investigated mechanisms of action discussed above, for marine compounds **175**–**188**, only an IC_50_ was reported and consolidated in Table 2, but their respective molecular mechanisms of action remained undetermined: asperterpenol A (**175**) [173]; cymatherelactone (**176**) [174]; dictyodendrin H (**177**) [175]; geranylphenazinediol (**178**) [176]; halomadurones C and D (**179**,**180**) [177]; lamellarin O (**39**) [53]; ircinianin lactams A (**181**,**182**) [178]; and polar steroids (**183**–**188**) [179]. 

Finally, marine bioprospecting resulting from deep sequencing of transcriptomes of marine organisms may ultimately enhance the search for new nervous system drug candidates, as demonstrated by a study of the adult polyp transcriptomes of two cold-water sea anemone species that revealed 15 new neurotoxin peptide candidates [181].

## 4. Marine Compounds with Miscellaneous Mechanisms of Action

Table 3 presents 2012–2013 preclinical pharmacological research of 69 marine compounds (**189**–**257**) with miscellaneous mechanisms of action; their structures are shown in Figure 3. Because comprehensive pharmacological characterization data for these compounds were unavailable, it was not possible to assign these compounds to a particular drug class.

Table 3 presents a pharmacological activity, an IC_50_, and a molecular mechanism of action for 36 marine natural products as reported in the peer-reviewed literature: astaxanthin (**189**) [182]; biselyngbyaside (**190**) [183]; *Callyspongia* sp. bisacetylenic alcohol (**191**) [184]; conicasterol E (**192**) [185]; 6”-debromohamacanthin A (**193**) [186]; dieckol (**194**) [187]; fructigenine A (**195**) [188]; geoditin A (**196**) [189]; gorgosterol (**197**) [190]; gracilioether B (**198**) [191]; gracilioether K (**199**) [192]; herdmanine K (**200**) [193]; hyrtioreticulin A (**201**) [194]; new Kunitz-type protease inhibitor InHVJ (**202**) [195]; jaspamide (**203**) [196]; latonduine A (**204**) [197]; leucettine L41 (**205**) [169]; manzamine A (**206**) [198]; nahuoic acid A (**207**) [199]; namalide (**208**) [200]; ningalins C and D (**209**,**210**) [201]; octaphlorethol A (**114**) [120]; petrosaspongiolide M (**211**) [202]; petrosiol A (**212**) [203]; phidianidine A (**213**) [204]; Poly-APS (**214**) [205]; *Pseudoceratina* sp. dibromotyrosine (**215**) [206]; pseudopterosin A (**216**) [207]; sargachromanol G (**217**) [208]; *S. graminifolium* polysaccharide (**218**) [209]; *S. patens* phloroglucinol (**219**) [210]; *S. xiamenensis* benzopyran (**220**) [211]; theonellasterol (**221**) [212]; toluquinol (**222**) [213]; and *U. lactuca* fatty acid (**223**) [214].

Also described in Table 3 is the pharmacological activity of 34 additional compounds. Albeit an IC_50_ for enzyme or receptor inhibition is provided, no mechanism of action studies were reported at the time of publication: alotaketal C (**224**) [215]; aspergentisyl A (**225**) [216]; *A. terreus* butyrolactone (**226**) [217]; caulerpine (**227**) [218]; conicasterol F (**228**) [219]; *D. avara* sesquiterpene (**229**) [220]; *D. gigantea* sterols (**230**,**231**) [221]; dysidavarone A (**232**) [222]; galvaquinone B (**233**) [223]; halicloic acids A and B (**234**,**235**) [224]; isochromophilone XI (**236**) [225]; leucettamols A and B (**237**,**238**) [226]; manadosterol A (**239**) [227]; marilines A1 and A2 (**240**,**241**) [228]; methyl sarcotroate B (**242**) [229]; *P. citrinum* sorbicillinoid (**243**) [230]; phosphoiodyn A (**244**) [231]; purpuroines A and D (**245**,**246**) [232]; santacruzamate A (**247**) [233]; sarcophytonolide N (**248**) [234]; sargassumol (**249**) [235]; sesquibastadin 1 (**250**) [236]; *S. glaucum* cembranoids (**251**–**253**) [237]; symplocin A (**254**) [238]; tsitsikammamine A derivative (**255**) [239]; *V. lanosa* bromophenol (**256**) **[240]**; and *X. testudinaria* fatty acid (**257**) [241].

## 5. Reviews on Marine Pharmacology

In 2012–2013, several reviews were published covering general and/or specific areas of marine preclinical pharmacology: (a) ***marine pharmacology and marine pharmaceuticals***: new marine natural products and relevant biological activities published in 2010 and 2011 [243,244]; natural products drug discovery as a continuing source of novel drug leads [245]; guiding principles for natural product drug discovery [246]; challenges and triumphs to genomic-based natural product discovery and pharmacology [247]; future of marine natural products drug discovery [248]; bioactive marine natural products from Antarctic and Arctic organisms [249]; biological activities of terpenes from the soft coral genus *Sarcophyton* [250]; pharmacologically active marine peptides from fish and shellfish [251]; preclinical pharmacology of marine diterpene glycosides [252]; bioactivity of fucoidan, a complex algal sulfated polysaccharide [253]; therapeutic application of marine fucanomics and galactanomics in drug development [254]; marine pharmacology of cosmopolitan brown alga *Cystoseira* genus secondary metabolites [255]; pharmacological activity of sulfated polysaccharides from marine algae [256]; biological activities and functions of halogenated organic molecules of red algae Rhodomelaceae [257]; pharmacological potential of marine cyanobacterial secondary metabolites [258]; pharmaceutical agents from filamentous marine cyanobacteria [259]; chemistry and preclinical pharmacology of sponge glycosides [260]; sea cucumbers as drug candidates [261]; bioactives from microalgal dinoflagellates [262]; the global marine pharmaceutical pipeline in 2017: U.S. Food and Drug Administration-approved compounds and those in Phase I, II and III of clinical development http://marinepharmacology.midwestern.edu/clinPipeline.htm; (b) ***antimicrobial marine pharmacology***: antimicrobial non-ribosomal peptides from abundant α-, γ- and δ-marine Proteobacteria classes [263]; marine bacteria as potential sources for compounds to overcome methicillin-resistant *Staphylococcus aureus* [264]; marine coral alkaloids and antibacterial activities [265]; marine fish and invertebrates as sources of antimicrobial peptides [266]; marine actinomycetes as an emerging resource for drug development [267]; chemistry and biological activity of marine *Bacillus* sp. secondary metabolites [268]; marine compounds with therapeutic potential in Gram-negative sepsis [269]; antimicrobial properties of tunichromes [270]; drug discovery from marine microbes [271]; (c) ***antiviral marine pharmacology***: marine natural products with anti-HIV activities in the last decade [272]; fucoidans as potential inhibitors of human immunodeficiency virus type 1 (HIV-1) [273]; discovery of potent broad spectrum antivirals derived from marine Actinobacteria [274]; algal lectins for prevention of HIV transmission [275]; (d) ***antiprotozoal, antimalarial, antituberculosis and antifungal marine pharmacology***: trypanocidal activity of marine natural products [276]; natural sesquiterpenes as lead compounds for the design of trypanocidal drugs [277]; antifungal compounds from marine fungi [278]; (e) ***immuno- and anti-inflammatory marine pharmacology***: immunoregulatory properties of bryostatin [279]; bioactive marine peptides as potential anti-inflammatory therapeutics [280]; anti-inflammatory soft coral marine natural products from Taiwan [281]; marine natural products with potential for the therapeutics of inflammatory diseases [282]; antioxidant properties of crude extracts and compounds from brown marine algae [283]; (f) ***cardiovascular and antidiabetic marine pharmacology***: oxidation of marine omega-3 supplements and human health [284]; marine peptides for prevention of metabolic syndrome [285]; antidiabetic effect of marine brown algae-derived phlorotannins [286]; marine bioactive peptides as potential antioxidants [287]; cardioprotective peptides from marine sources [288]; antioxidant and antidiabetic pharmacology of fucoxantin [289]; marine-derived bioactive peptides as new anticoagulants [290]; (g) ***nervous system marine pharmacology***: marine neurotoxins, structures, molecular targets and pharmacology [291]; the phosphatase inhibitor okadaic acid as a tool to identify phosphoepitopes relevant to neurodegeneration [292]; marine toxins and drug discovery targeting nicotinic acetylcholine receptors [293]; marine-derived marine secondary metabolites and neuroprotection [294]; cone snail polyketides active in neurological assays [295]; and (h) ***miscellaneous molecular targets and uses***: small-molecule inhibitors of clinically validated protein and lipid kinases of marine origin [296]; natural products as kinase inhibitors [297]; marine natural products with protein tyrosine phosphatase 1B activity [298]; current development strategies for marine conotoxins and their mimetics as therapeutic leads [299]; therapeutic potential of novel conotoxins reported in 2007–2011 [300]; computational studies of marine toxins targeting ion channels [301]; marine invertebrates as sources of skeletal proteins for bone regeneration [302]; marine algal compounds in cosmeceuticals [303]; and marine sponge steroids as nuclear receptor ligands [304]. 

## 6. Conclusions

The purpose of the current marine pharmacology review was to continue the marine *preclinical* pharmacology pipeline review series that was initiated in 1998 [1,2,3,4,5,6,7,8] by consolidating preclinical marine pharmacological research published during 2012–2013 in the global literature. The large number of peer-reviewed publications we have reviewed demonstrates that the global research effort involved chemists and pharmacologists from 43 countries, namely, Argentina, Australia, Austria, Belgium, Brazil, Canada, Chile, China, Colombia, Egypt, Fiji, France, French Polynesia, Germany, Greece, India, Indonesia, Ireland, Israel, Italy, Japan, Malaysia, Mexico, Morocco, the Netherlands, New Zealand, Norway, Pakistan, Panama, Papua New Guinea, Russian Federation, Saudi Arabia, Slovenia, South Africa, South Korea, Spain, Sri Lanka, Switzerland, Taiwan, Thailand, United Kingdom, Vietnam, and the United States. Thus, during 2012–2013 the marine *preclinical* pharmaceutical pipeline continued to provide novel pharmacological lead compounds that enriched the marine *clinical* pharmaceutical pipeline. Currently, the *clinical* pharmaceutical pipeline consists of 6 pharmaceuticals approved by the U.S. Food and Drug Administration, and 29 compounds in Phase I, II and III of clinical pharmaceutical development, as shown at a dedicated website: http://marinepharmacology.midwestern.edu/clinPipeline.htm.

## Figures and Tables

**Table 1:** Marine pharmacology in 2012–2013: marine compounds with antibacterial, antifungal, antituberculosis, antiprotozoal, antiviral and anthelmintic activities.

Drug Class	Compound/Organism ^a^	Chemistry	Pharmacologic Activity	IC_50_^b^	MMOA ^b^	Country ^c^	References
Antibacterial	anthracimycin (**1**)/bacterium	Polyketide ^d^	*B. anthracis* & *S. aureus* inhibition	0.03–0.06 μg/mL ^+^	DNA/RNA inhibition	USA	[31]
Antibacterial	chrysophaentins (**2**,**3**)/alga	Shikimate ^h^	Gram-negative & -positive bacterial inhibition	27–84 μM ^+^	Competitive inhibition of FtsZ GTP-binding site	ESP, USA	[32]
Antibacterial	merochlorin A (**4**)/bacterium	Terpenoid ^e^	*C. dificile* & *S. aureus* strains inhibition	0.3–2 μg/mL ^+^	DNA, RNA, protein & cell wall synthesis inhibition	USA	[33]
Antibacterial	aflatoxin B2b (**5**)/fungus	Polyketide ^d^	*B. subtilis* & *E. aerogenes* inhibition	1.7, 1.1 μM ^+^	Undetermined	CHN	[34]
Antibacterial	ageloxime B (**6**)/sponge	Alkaloid/terpenoid ^e^	*S. aureus* inhibition	7.2–9.2 μg/mL *	Undetermined	CHN, USA	[35]
Antibacterial	*Alternaria* sp. anthraquinones (**7**–**9**)/fungus	Polyketide ^d^	*E. coli* & *V. parahemolyticus* inhibition	0.62–5 μM ^+^	Undetermined	CHN	[36]
Antibacterial	antimycin B2 (**10**)/bacterium	Shikimate/Polyketide ^d^	*L. hongkongensis* inhibition	8 μg/mL ^+^	Undetermined	CHN	[37]
Antibacterial	*Aspergillus* sp. (−)sydonol (**11**)/fungus	Terpenoid ^e^	*S. albus* & *M. tetragenus* inhibition	1.2–5 μg/mL ^+^	Undetermined	CHN, NLD	[38]
Antibacterial	axistatins 1–3 (**12**–**14**)/sponge	Alkaloid/terpenoid ^e^	*C. neoformans* & *S. aureus* inhibition	1–4 μg/mL ^+^	Undetermined	AUS, USA	[39]
Antibacterial	bromophycoic acid A & E (**15**,**16**)/alga	Terpenoid ^e^	*S. aureus* & *E. faecilis* inhibition	1.6 μg/mL ^+^	Undetermined	FJI, USA	[40]
Antibacterial	cadeolides C–F (**17**–**20**)/tunicate	Shikimate ^h^	*S. aureus* inhibition	0.13–3 μg/mL ^+^	Undetermined	S. KOR	[41]
Antibacterial	cadiolides E–I (**21**–**23**)/ascidian	Shikimate ^h^	*S. aureus* & *B. subtilis* inhibition	0.8–12 μg/mL ^+^	Undetermined	S. KOR	[42]
Antibacterial	citreamicin *θ* A & B (**24**,**25**)/bacterium	Polyketide ^d^	*S. aureus* inhibition	0.25–1 μg/mL *	Undetermined	CHN, SAU	[43]
Antibacterial	communol A & F (**26**,**27**)/fungus	Polyketide ^d^	*E. coli* inhibition	4.1, 6.4 μg/mL ^+^	Undetermined	CHN	[44]
Antibacterial	*D. spiralis* dolabellanes (**28**,**29**)/alga	Terpenoid ^e^	*S. aureus* inhibition	2–8 μg/mL ^+^	Undetermined	GRC, ESP, UK	[45]
Antibacterial	enhygrolide A (**30**)/bacterium	Shikimate ^h^	*A. cristallopoietes* inhibition	4 μg/mL ^+^	Undetermined	DEU	[46]
Antibacterial	eudistomin Y11 (**31**)/ascidian	Alkaloid ^f^	*B. subtilis* & *S. typhimurium* inhibition	3.12 μg/mL ^+^	Undetermined	S. KOR	[47]
Antibacterial	fradimycin B (**32**)/bacterium	Polyketide ^d^	*S. aureus* inhibition	2.0 μg/mL ^+^	Undetermined	CHN	[48]
Antibacterial	*Haliclona* diAPS (**33**–**35**)/sponge	Alkaloid ^f^	*M. luteus* inhibition	3.1 μg/mL ^+^	Undetermined	S. KOR	[49]
Antibacterial	hyrtimomine D (**36**)/sponge	Alkaloid ^f^	*S. aureus* inhibition	4 μg/mL ^+^	Undetermined	JPN	[50]
Antibacterial	ianthelliformisamine A (**37**)/sponge	Alkaloid ^f^	*P. aeruginosa* inhibition	6.8 μM	Undetermined	AUS	[51]
Antibacterial	kocurin (**38**)/bacterium	Peptide ^f^	MR *S. aureus* inhibition	0.25 μg/mL ^+^	Undetermined	ESP, USA	[52]
Antibacterial	lamellarin O (**39**)/sponge	Alkaloid ^f^	*B. subtilis* inhibition	2.5 μM	Undetermined	AUS	[53]
Antibacterial	*Laurencia* sesquiterpenes (**40**–**42**)/alga	Terpenoid ^e^	*E. coli* & *S. aureus* inhibition	5–7 μg/disk ^++^	Undetermined	CHN, USA	[54]
Antibacterial	lobophorin H (**43**)/bacterium	Terpenoid glycoside	*B. subtilis* inhibition	1.57 μg/mL ^+^	Undetermined	CHN	[55]
Antibacterial	marthiapeptide A (**44**)/bacterium	Peptide ^f^	*M. luteus* & *B. thuringiensis* inhibition	2.0 μg/mL *	Undetermined	CHN	[56]
Antibacterial	napyradiomycin A1 & B3 (**45**,**46**)/bacterium	Terpenoid/polyketide ^d^	*S. aureus* inhibition	0.5–2 μg/mL ^+^	Undetermined	CHN	[57,58]
Antibacterial	*Nigrospora* sp. anthraquinones (**47**,**48**)/fungus	Polyketide ^d^	*E. coli* & *S. aureus* inhibition	0.6–0.7 μM ^+^	Undetermined	CHN	[59]
Antibacterial	ohmyungsamycin A (**49**)/bacterium	Peptide ^f^	*B. subtilis* inhibition	4.28 μM ^+^	Undetermined	S. KOR	[60]
Antibacterial	penicifuran A (**50**)/fungus	Shikimate ^h^	*S. albus* inhibition	3.1 μM ^+^	Undetermined	CHN	[61]
Antifungal	crambescidin-816 (**51**)/sponge	Alkaloid ^f^	*S. cerevisiae* growth inhibition	1 μM ^+^	G2/M cell cycle arrest and apoptosis	ESP, FRA	[62]
Antifungal	neothyonidioside (**52**)/sea cucumber	Terpenoid glycoside	*S. cerevisiae* inhibition	1 μM ^+^	Binding to plasma membrane sterols	NZL	[63]
Antifungal	ageloxime B (**6**)/sponge	Alkaloid/terpenoid	*C. neoformans* inhibition	4.9 μg/mL *	Undetermined	CHN, USA	[35]
Antifungal	aurantoside K (**53**)/sponge	Polyketide/alkaloid glycoside	*C. albicans* inhibition	1.95 μg/mL ^+^	Undetermined	FJI	[64]
Antifungal	caulerprenylol B (**54**)/alga	Terpenoid ^e^	*C. glabrata* & *C. neoformans* inhibition	4.0 μg/mL ^+^	Undetermined	CHN	[65]
Antifungal	didymellamide A (**55**)/fungus	Alkaloid ^f^	*C. albicans* inhibition	3.1 μg/mL ^+^	Undetermined	JPN	[66]
Antifungal	hippolachnin A (**56**)/sponge	Polyketide ^d^	*T. rubrum, M. gypseum* & *C. neoformans* inhibition	0.41 μM ^+^	Undetermined	CHN	[67]
Antifungal	holotoxins F & G (**57**,**58**)/sea cucumber	Terpenoid glycoside	*C. albicans*, *Microsporum* & *Cryptococcus* inhibition	1.4–5.8 μM ^+^	Undetermined	CHN, DEU	[68]
Antifungal	hyrtimomine D & E (**36**,**59**)/sponge	Alkaloid ^f^	*C. albicans* & *C. neoformans* inhibition	4–16 μg/mL ^+^	Undetermined	JPN	[50]
Antifungal	nagelamide Z (**60**)/sponge	Alkaloid ^f^	*C. albicans* inhibition	0.25 μg/mL *	Undetermined	JPN	[69]
Antifungal	woodylide A (**61**)/sponge	Polyketide ^d^	*C. neoformans* inhibition	3.7 μg/mL *	Undetermined	CHN	[70]
Antiprotozoal	araplysillin I (**62**)/sponge	Alkaloid ^f^	*P. falciparum* FcB1 & 3D7 strain inhibition	4.5 μM	Undetermined	AUS, DEU, FJI, FRA	[71]
Antiprotozoal	ascidiathiazone A (**63**)/ascidian	Alkaloid ^f^	*P. falciparum* K1 strain inhibition	3.3 μM	Undetermined	NZL, CHE	[72]
Antiprotozoal	axidjiferosides A–C (**64**–**66**)/sponge	Glycosphingolipid	*P. falciparum* FcB1strain inhibition	0.53 μM	Undetermined	FRA	[73]
Antiprotozoal	cytosporone E (**67**)/fungus	Polyketide ^d^	*P. falciparum* inhibition	13 μM **	Undetermined	USA	[74]
Antiprotozoal	dicerandrol D (**68**)/fungus	Polyketide ^d^	*P. falciparum* 3D7 strain inhibition	0.6 μM	Undetermined	CHN, TWN, USA	[75]
Antiprotozoal	dihydroingenamine D (**69**)/sponge	Alkaloid ^f^	*P. falciparum* D6 & W2 strain inhibition	57–72 ng/mL	Undetermined	USA	[76]
Antiprotozoal	19-hydroxypsammaplysin E (**70**)/sponge	Alkaloid ^f^	*P. falciparum* 3D7strain inhibition	6.4 μM	Undetermined	AUS, IDN	[77]
Antiprotozoal	kabiramide L (**71**)/sponge	Polyketide ^d^	*P. falciparum* K1 strain inhibition	2.6 μM	Undetermined	THAI, AUT	[78]
Antiprotozoal	meridianin C & G (**72**,**73**)/tunicate	Alkaloid ^f^	*P. falciparum* D6 & W2 strain inhibition	4.4–14.4 μM	Undetermined	IND	[79]
Antiprotozoal	orthidine F (**74**)/ascidian	Alkaloid ^f^	*P. falciparum* K1 strain inhibition	0.90 μM	Undetermined	CHE, NZL	[80]
Antiprotozoal	plakortide U (**75**)/sponge	Polyketide ^d^	*P. falciparum* FcM29 strain inhibition	0.8 μM	Undetermined	FRA, ITA	[81]
Antiprotozoal	thiaplakortone A (**76**)/sponge	Alkaloid ^f^	*P. falciparum* 3D7 & Dd2 strain inhibition	0.006–0.051 μM	Undetermined	AUS	[82]
Antiprotozoal	tsitikammamine C (**77**)/sponge	Alkaloid ^f^	*P. falciparum* 3D7 & Dd2 strain inhibition	13 & 18 nM	Undetermined	AUS	[83]
Antiprotozoal	urdamycinone E (**78**)/bacterium	Polyketide ^d^	*P. falciparum* K1 strain inhibition	0.05 μg/mL	Undetermined	THAI	[84]
Antiprotozoal	almiramide (**79**,**80**)/bacterium	Peptide ^f^	*T. brucei* inhibition	0.4–3.5 μM	Glycosome function inhibition	USA	[85]
Antiprotozoal	diazepinomicin (**81**)/bacterium	Alkaloid/terpenoid	*T. brucei* inhibition	13.5 μM	Rhodesain inhibition	EGY, DEU	[86]
Antiprotozoal	(−)-elatol (**82**)/alga	Terpenoid ^e^	*T. cruzi* inhibition	1.5–3 μM *	Mitochondrial disfunction	BRA	[87]
Antiprotozoal	ascidiathiazone A (**63**)/ascidian	Alkaloid ^f^	*T. b. rhodesiense* inhibition	3.1 μM	Undetermined	NZL, CHE	[72]
Antiprotozoal	coibacin A (**83**)/bacterium	Polyketide ^d^	*L. donovani* inhibition	2.4 μM	Undetermined	USA, PAN	[88]
Antiprotozoal	cristaxenicin A (**84**)/gorgonian	Terpenoid ^e^	*T. congolense* & *L. amazonensis* inhibition	0.25 & 0.088 μM	Undetermined	JPN	[89]
Antiprotozoal	manadoperoxide B analogues (**85**,**86**)/sponge	Polyketide ^d^	*T. b. rhodesiense* inhibition	3–11 ng/mL	Undetermined	ITA, IDN, CHE, IRL	[90]
Antituberculosis	asperterpenoid A (**87**)/fungus	Terpenoid ^e^	*M. tuberculosis* PTP inhibition	2.2 μM	Undetermined	CHN	[91]
Antituberculosis	brevianamide S (**88**)/fungus	Alkaloid ^f^	BCG inhibition	6.25 μg/mL ^+^	Undetermined	AUS, CHN	[92]
Antituberculosis	lobophorin G (**89**)/bacterium	Terpenoid ^e^ glycoside	BCG inhibition	1.56 μg/mL ^+^	Undetermined	CHN	[93]
Antituberculosis	neamphamide B (**90**)/sponge	Peptide ^f^	*M. bovis* inhibition	1.56 μg/mL ^+^	Undetermined	JPN	[94]
Antituberculosis	*S. flava* diterpenes (**91**,**92**)/sponge	Terpenoid ^e^	*M. tuberculosis* H37Rv inhibition	15, 32 μg/mL ^+^	Undetermined	USA	[95]
Antituberculosis	urdamycinone E (**78**)/bacterium	Polyketide ^d^	*M. tuberculosis* H37Ra inhibition	3.13 μg/mL ^+^	Undetermined	THAI	[84]
Antiviral	halistanol sulfates (**93**,**94**)/sponge	Terpenoid ^f^	Human *Herpes simplex* virus-1 inhibition	0.5–12.2 μg/mL	Attachment & penetration inhibition	ARG, BRA	[96]
Antiviral	*L. arboreum* metabolites (**95**–**97**)/soft coral	Terpenoid/sphingolipid	HIV-1 protease inhibition	4.8–7.2 μM *	Molecular docking & HIV-1 protease receptor	ZAF	[97]
Antiviral	manoalide (**98**)/sponge	Terpenoid ^e^	Hepatitis C virus inhibition	15–70 μM	RNA helicase and ATPase inhibition	JPN	[98]
Antiviral	*N. aculeata* metabolites (**99**,**100**)/alga	Polyketide ^d^	Human rhinoviruses 2 & 3 inhibition	2.5–7.1 μg/mL	Cytopathic effect inhibition	S. KOR	[99]
Antiviral	stachybotrin D (**101**)/fungus	Alkaloid/terpenoid	HIV-1 replication inhibition	8.4 μM	Reverse transcriptase inhibition	CHN	[100]
Antiviral	streptoseolactone (**102**)/bacterium	Terpenoid ^f^	Neuraminidase inhibition	3.9 μM	Noncompetitive inhibition	CHN	[101]
Antiviral	asperterrestide A(**103**)/fungus	Peptide ^f^	H3N2 influenza virus inhibition	8.1 μM	Undetermined	CHN	[102]
Antiviral	*Cladosporium* sp. alkaloids (**104**,**105**)/fungus	Alkaloid ^f^	H1N1 influenza virus inhibition	82–85 μM	Undetermined	CHN	[103]
Antiviral	isorhodoptilometrin-1-methyl ether (**106**)/fungus	Polyketide ^d^	Hepatitis C NS3/4A protease inhibition	>1 ng/mL *	Undetermined	EGY	[104]
Antiviral	massarilactone H (**107**)/fungus	Polyketide ^d^	Influenza virus neuraminidase inhibition	8.2 μM	Undetermined	CHN, MYS	[105]
Antiviral	pyronepolyene C-glucoside (**108**)/fungus	Polyketide ^d^	H1N1 influenza virus inhibition	91.5 μM	Undetermined	CHN	[106]
Antiviral	*S. candidula* sterol (**109**,**110**)/soft coral	Terpenoid/sphingolipid	H5N1 avian influenza virus inhibition	1 ng/mL *	Undetermined	EGY	[107]
Antiviral	*S. vulgare* glycolipid (**111**)/alga	Glycolipid	Human herpes simplex virus-1 & 2 inhibition	<50 μg/mL	Undetermined	BRA	[108]
Anthelmintic	echinosides A & B (**112**,**113**)/sea cucumber	Terpenoid glycoside	*S. mansoni* worm lethality	0.19, 0.27 μg/mL ^+++^	Undetermined	EGY	[109]

(^a^) **Organism**: *Kingdom Animalia*: ascidian (Phylum Chordata), gorgonian, coral (Phylum Cnidaria), sea cucumber (Phylum Echinodermata), sponge (Phylum Porifera); *Kingdom Monera*: bacterium (Phylum Cyanobacteria); *Kingdom Fungi*: fungus; *Kingdom Plantae:* alga; (^b^) **IC_50_**: concentration of a compound required for 50% inhibition in vitro, *: estimated IC_50_, **: IC_90_, ^+^: MIC: minimum inhibitory concentration, ^++^: MID: minimum inhibitory concentration per disk; ^+++^: LC_50_: concentration of a compound required for 50% lethality; **MMOA**: molecular mechanism of action; (^c^) **Country**: ARG: Argentina; AUS: Australia; AUT: Austria; BRA: Brazil; CHE: Switzerland; CHN: China; DEU: Germany; EGY: Egypt; ESP: Spain; FJI: Fiji; FRA: France; GRC: Greece; IDN: Indonesia; IND: India; IRL: Ireland; ITA: Italy; JPN: Japan; MYS: Malaysia; NLD: The Netherlands; NZL: New Zealand; PAN: Panama; SAU: Saudi Arabia; S. KOR: South Korea; THAI: Thailand; TWN: Taiwan; UK: United Kingdom; ZAF: S. Africa; **Chemistry**: (^d^) Polyketide; (^e^) Terpene; (^f^) Nitrogen-containing compound; (^g^) Polysaccharide, (^h^) Shikimate; **Abbreviations**: BCG: Bacille Calmette-Guérin; diAPS: dialkylpyridinium; MR: methicillin-resistant.

**Table 2:** Marine pharmacology in 2012–2013: marine compounds with antidiabetic and anti-inflammatory activity; and affecting the immune and nervous system.

Drug Class	Compound/Organism ^a^	Chemistry	Pharmacological Activity	IC_50_^b^	MMOA ^c^	Country ^d^	References
Antidiabetic	octaphlorethol A (**114**)/alga	Polyketide ^e^	Increased glucose uptake in rat myoblast cells	50 μM *	Glucose transporter 4 translocation	S. KOR	[120]
Anti-inflammatory	apo-9′-fucoxanthinone (**115**)/alga	Terpenoid ^f^	Macrophage TNF-α, IL-6 & 12 expression inhibition	5–14 μM	MAPK pathway inhibition	S. KOR	[121]
Anti-inflammatory	astaxanthin (**116**)/alga	Terpenoid ^f^	Macrophage cytokine inhibition	10 μM *	SHP-1 restoration	ITA	[122]
Anti-inflammatory	bengamide A & B (**117**,**118**)/sponge	Alkaloid ^g^	Macrophage TNF-α & IL-6 inhibition	0.5 μM *	IĸBα phosphorylation inhibition	USA	[123]
Anti-inflammatory	bis-*N*-norgliovictin (**119**)/fungus	Alkaloid ^g^	Macrophage TNF-α, IL1-6, MCP-1 release inhibition in vitro	0.5 μg/mL *	Inflammatory gene inhibition	CHN	[124]
Anti-inflammatory	6,6′-bieckol (**120**)/alga	Polyketide ^e^	Macrophage TNF-α & IL-6 expression inhibition	25 μM *	Inhibition of NFκB	S. KOR, USA	[125]
Anti-inflammatory	coibacin B (**121**)/bacterium	Polyketide ^e^	Macrophage NO inhibition	5 μM	iNOS, TNF-α, IL-1, IL-6 transcription inhibition	USA, PAN	[88]
Anti-inflammatory	11-*epi*-sinulariolide acetate (**122**)/soft coral	Terpenoid ^f^	Macrophage COX-2 & IL-8 expression inhibition	10 μM	Ca^2+^ signaling inhibition	TWN	[126]
Anti-inflammatory	honaucin A (**123**)/bacterium	Polyketide ^e^	Macrophage NO inhibition	4 μM	iNOS, TNF-α, IL-1, IL-6 transcription inhibition	USA, PAN	[127]
Anti-inflammatory	*Hymeniacidon* sp. amphilectanes (**124**,**125**)/sponge	Terpenoid ^f^	Brain microglia TXB_2_ inhibition	0.2 μM	SOX independent & COX dependent	USA	[128]
Anti-inflammatory	largazole (**126**)/bacterium	Peptide ^g^	Modulation of human RA synovial fibroblasts in vitro	5 μM *	Enhanced HDAC6 & ICAM-1	USA	[129]
Anti-inflammatory	lemnalol (**127**)/soft coral	Terpenoid ^f^	In vivo arthritis inhibition	30 mg/kg*	iNOS, COX-2 and c-Fos expression inhibition	TWN	[130]
Anti-inflammatory	neoechinulin A (**128**)/fungus	Alkaloid^g^	Macrophage PGE_2_ and NO expression inhibition	25–50 μM *	Inhibition of NFκB & MAPK	S. KOR; CHN	[131]
Anti-inflammatory	penstyrylpyrone (**129**)/fungus	Shikimate/polyketide	Macrophage NO, PGE_2_, IL1β inhibition	9.3–13.5 μM	PTP1B inhibition	S. KOR	[132]
Anti-inflammatory	perthamide C (**130**)/sponge	Peptide ^g^	Carrageenan-induced paw edema inhibition	ND	Induction of proteome changes	ITA	[133]
Anti-inflammatory	R-prostaglandins (**131**,**132**)/soft coral	Polyketide ^e^	Topical inflammation inhibition	ND	PMN elastase inhibition	COL	[134]
Anti-inflammatory	sinularin (**133**)/soft coral	Terpenoid ^f^	Carrageenan-induced spinal neuroinflammation inhibition	0.1 μM *	iNOS & COX-2 inhibition	TWN	[135]
Anti-inflammatory	swinhosterol B (**134**)/sponge	Terpenoid ^f^	Lymphocyte release of IL-10	10 μM *	Pregnane-X-receptor agonist	ITA, FRA	[136]
Anti-inflammatory	*A. polyacanthus* steroids (**135**,**136**)/starfish	Terpenoid ^f^	Bone marrow-derived dendritic cells IL-6 and TNF-α inhibition	1.8–7.0 μM	Undetermined	S. KOR, VNM	[137]
Anti-inflammatory	barettin (**137**)/sponge	Alkaloid ^g^	Macrophage anti-inflammatory IL-10 release in vitro	50 μg/mL	Undetermined	NOR	[138]
Anti-inflammatory	briarenolide F (**138**)/octocoral	Terpenoid ^f^	Neutrophil superoxide inhibition	3.82 μg/mL	Undetermined	TWN	[139]
Anti-inflammatory	*Callyspongia* sp. diketopiperazine (**139**)/sponge	Peptide ^g^	Macrophage IL1β release inhibition in vitro	5 μg/mL *	Undetermined	CHN	[140]
Anti-inflammatory	6-*epi*-cladieunicellin F (**140**)/octocoral	Terpenoid ^f^	Neutrophil superoxide and elastase inhibition	10 μM *	Undetermined	TWN	[141]
Anti-inflammatory	crassarosteroside A (**141**)/soft coral	Terpenoid glycoside ^f^	Macrophage iNOS protein inhibition	10 μM *	Undetermined	TWN	[142]
Anti-inflammatory	cystodione A (**142**)/alga	Terpenoid ^f^	Radical-scavenging and macrophage TNF-α inhibition in vitro	8–22 μM *	Undetermined	ESP, MAR	[143]
Anti-inflammatory	densanins A & B (**143**,**144**)/sponge	Alkaloid ^g^	Macrophage NO release inhibition	1–2.1 μM	Undetermined	S. KOR	[144]
Anti-inflammatory	dissesterol (**145**)/soft coral	Terpenoid ^f^	Bone marrow dendritic cells IL-12 release inhibition	4 μM	Undetermined	S. KOR, VNM	[145]
Anti-inflammatory	echinohalimane A (**146**)/gorgonian	Terpenoid ^f^	Neutrophil elastase inhibition	0.38 μg/mL	Undetermined	TWN	[146]
Anti-inflammatory	eunicidiol (**147**)/gorgonian	Terpenoid ^f^	PMA-induced mouse ear edema inhibition	100 μg/ear	Undetermined	CAN	[147]
Anti-inflammatory	flexibilisolide C (**148**)/soft coral	Terpenoid ^f^	Macrophage COX-2 & iNOS expression inhibition	10 μM *	Undetermined	TWN	[148]
Anti-inflammatory	flexibilisquinone (**149**)/soft coral	Terpenoid ^f^	Macrophage COX-2 & iNOS expression inhibition	10–20 μM *	Undetermined	TWN	[149]
Anti-inflammatory	lobocrassin F (**150**)/soft coral	Terpenoid ^f^	Neutrophil elastase release inhibition	6.3 μM *	Undetermined	TWN	[150]
Anti-inflammatory	perthamide J (**151**)/sponge	Peptide ^g^	Carrageenan-induced paw edema reduction	0.3 mg/kg *	Undetermined	ITA, FRA	[151]
Anti-inflammatory	pseudoalteromone A (**152**)/bacterium	Terpenoid ^f^	Neutrophil elastase inhibition	10 μg/mL *	Undetermined	TWN	[152]
Anti-inflammatory	sarcocrassocolide M (**153**)/soft coral	Terpenoid ^f^	Macrophage COX-2 & iNOS expression inhibition	10 μM *	Undetermined	TWN	[153]
Anti-inflammatory	sclerosteroids K & M (**154**,**155**)/soft coral	Terpenoid ^f^	Macrophage COX-2 & iNOS expression inhibition	10 μM *	Undetermined	TWN	[154]
Anti-inflammatory	seco-briarellinone (**156**)/octocoral	Terpenoid ^f^	Macrophage NO release inhibition	4.7 μM	Undetermined	PAN	[155]
Anti-inflammatory	sinularioside (**157**)/soft coral	Glycolipid	Macrophage NO release inhibition	30 μM *	Undetermined	ITA	[156]
Immune system	lobocrassin B (**158**)/soft coral	Terpenoid ^f^	Dendritic cell activation inhibition	39 μM *	NF-κB translocation and TNF-α release inhibition	TWN	[157]
Immune system	penicacid B(**159**)/fungus	Polyketide ^e^	T lymphocyte proliferation inhibition	0.23–20 μM	IMPDH inhibition	CHN	[158]
Nervous system	APETx2 peptide (**160**)/sea anemone	Peptide ^g^	ASIC3 inhibition	61 nM	*N*- and *C*- termini truncation decrease inhibition	AUS	[159]
Nervous system	asteropsin A (**161**)/sponge	Peptide ^g^	Enhancement of neuronal Ca^2+^ influx	14 nM	No binding with VGSC site 2	S. KOR, USA	[160]
Nervous system	BcsTx peptides (**162**,**163**)/sea anemone	Peptide ^g^	rKv1.1 inhibition	0.02–80 nM	Potassium influx inhibition	BRA, BEL	[161]
Nervous system	*C. consors* peptide (**164**)/cone snail	Peptide ^g^	Muscle relaxation induction	0.15 μM	Na_v_1.4 & Na_v_1.2 channel inhibition	BEL, FRA, CHE, CHL, DEU, NLD,	[162]
Nervous system	*C. magnificus* conotoxin MfVIA(**165**)/cone snail	Peptide ^g^	Neuronal Na^+^ current inhibition	95 nM	Na_v_1.8 and Na_v_1.4 channel inhibition	AUS	[163]
Nervous system	*C. regius* conotoxin RegIIA (**166**)/cone snail	Peptide ^g^	ACH-current inhibition	33 nM	Α2β2 ACH receptor	AUS, DEU, USA	[164]
Nervous system	*C. regularis* peptide (**167**)/cone snail	Peptide ^g^	Antinociceptive activity	0.85 mg/kg *	Ca_v_2.2 channel inhibition	MEX	[165]
Nervous system	convolutamydine A (**168**)/bryozoa	Alkaloid ^g^	Antinociceptive activity	1 mg/kg	Cholinergic, opioid and nitric oxide	BRA	[166]
Nervous system	*H. crispa* polypeptides (**169**)/sea anemone	Peptide ^g^	Antinociceptive and analgesic activity in vivo	0.01–0.1 mg/kg *	Inhibition of TRPV1 vanilloid 1 receptor	RUS	[167]
Nervous system	ianthellamide A (**170**)/sponge	Alkaloid ^g^	Increased kynurenic acid in vivo	200 mg/kg *	Kynurenine 3- hydroxylase inhibition	AUS	[168]
Nervous system	leucettamine B (**171**)/sponge	Alkaloid ^g^	Reduction of neurodegeneration in brain slices by analog leucettine L41	0.6–4.1 μM	Dual tyrosine phosphorylation kinase inhibition	FRA, UK, USA	[169]
Nervous system	pulchranin A (**172**)/sponge	Alkaloid ^g^	TRPV1 receptor inhibition	41.2 μM	Ca^2+^ response inhibition	RUS, S. KOR	[170]
Nervous system	serinolamide B (**173**)/bacterium	Alkaloid ^g^	CB_1_ & CB_2_ binding	**	cAMP accumulation inhibition	USA	[171]
Nervous system	arigsugacin I (**174**)/fungus	Terpenoid ^f^	acetylcholinesterase inhibition	0.64 μM	Undetermined	CHN	[172]
Nervous system	asperterpenol A (**175**)/fungus	Terpenoid ^f^	acetylcholinesterase inhibition	2.3 μM	Undetermined	CHN	[173]
Nervous system	cymatherelactone (**176**)/alga	Polyketide ^e^	voltage-gated sodium channel inhibition	16 μM	Undetermined	USA	[174]
Nervous system	dictyodendrin H (**177**)/sponge	Alkaloid ^g^	BACE inhibition	1 μM	Undetermined	AUS	[175]
Nervous system	geranylphenazinediol (**178**)/bacterium	Alkaloid ^g^	acetylcholinesterase inhibition	2.62 μM	Undetermined	DEU	[176]
Nervous system	halomadurones C & D (**179**,**180**)/bacteria	Terpenoid ^e^	Nrf2-ARE activation	3.7 μM *	Undetermined	USA	[177]
Nervous system	lamellarin O (**39**)/sponge	Alkaloid ^g^	BACE inhibition	<10 μM	Undetermined	AUS	[53]
Nervous system	*Psammocinia* sp. ircinianin lactams (**181**,**182**)/sponge	Terpenoid ^f^	A3 GlyR potentiation	8.5 μM	Undetermined	AUS, DEU	[178]
Nervous system	starfish polar steroids (**183**–**188**)/starfish	Terpenoid ^f^	Neuritogenic and neuroprotective	1–100 nM	Undetermined	RUS	[179]

(^a^) **Organism**: *Kingdom Animalia*: coral and sea anemone (Phylum Cnidaria); starfish (Phylum Echinodermata); cone snail (Phylum Mollusca); sponge (Phylum Porifera); *Kingdom Fungi*: fungus; *Kingdom Plantae:* alga; *Kingdom Monera*: bacterium; (^b^) **IC_50_**: concentration of a compound required for 50% inhibition, *: apparent IC_50_, **: *K*i 16.4 and 2 μM, respectively; (^c^) **MMOA**: molecular mechanism of action; (^d^) **Country**: AUS: Australia; BEL: Belgium; BRA: Brazil; CHE: Switzerland; CHL: Chile; CHN: China; COL: Colombia; DEU: Germany; ESP: Spain; FRA: France; ITA: Italy; MAR: Morocco; MEX: Mexico; NLD: Netherlands; NOR: Norway; PAN: Panama; RUS: Russian Federation; S. KOR: South Korea; TWN: Taiwan; UK: United Kingdom; VNM: Vietnam; **Chemistry**: (^e^) Polyketide; (^f^) Terpene; (^g^) Nitrogen-containing compound; (^h^) polysaccharide. **Abbreviations**: ASIC3: pH-sensitive sodium ion channel 3; BACE: protease β-secretase; COX: cyclooxygenase; GlyR: glycine-gated chloride channel receptor; HDAC6: class II, histone deacetylase 6; ICAM: intercellular adhesion molecule-1; iNOS: inducible nitric oxide synthase; IMPDH: inosine 5′-monophosphate dehydrogenase; MAPK: mitogen-activated protein kinase pathway; NO: nitric oxide; Nrf2-ARE: nuclear transcription factor E2-related factor antioxidant response element; PTP1B: tyrosine protein phosphatase 1B; rKv1.1: voltage-gated potassium channel Kv subfamily; SHP1: SHP-1 protein tyrosine phosphatase; SOX: superoxide; TRPV1: transient receptor potential cationic channel of subfamily V.

**Table 3:** Marine pharmacology in 2012–2013: marine compounds with miscellaneous mechanisms of action.

Compound/Organism ^a^	Chemistry	Pharmacological Activity	IC_50_^b^	MMOA ^c^	Country ^d^	References
astaxanthin (**189**)/alga	Terpenoid ^f^	Human sperm capacitation	2 μM *	Increased tyrosine phosphorylation	ITA	[182]
astaxanthin (**189**)/alga	Terpenoid ^f^	Apoptosis reduction in retinal ganglion cells	2 μM	H_2_O_2_ inhibition	CHN	[242]
biselyngbyaside (**190**)/bacterium	Polyketide ^e^	Osteoclast apoptosis induction	30 nM *	c-Fos and NFATc1 inhibition	JPN	[183]
*Callyspongia* sp. bisacetylenic alcohol (**191**)/sponge	Polyketide ^e^	Lymphatic endothelial cell proliferation inhibition	0.11 μM	Cell cycle arrest	JPN, NLD	[184]
conicasterol E (**192**)/sponge	Terpenoid ^f^	Bile acid detoxification	10 μM *	Farnesoid and pregnane receptor activity modulation	ITA, PYF	[185]
6′′-debromohamacanthin A (**193**)/sponge	Alkaloid ^g^	Angiogenesis inhibition	14.8 μM	PI3K/AKT/mTOR signaling inhibition	CAN, S. KOR	[186]
dieckol (**194**)/alga	Polyketide ^e^	Inhibition of melanin synthesis	>120 µM *	Cellular tyrosinase inhibition	S. KOR	[187]
fructigenine A (**195**)/fungus	Alkaloid ^g^	PTP1B inhibition	10.7 μM	Noncompetitive inhibition	S. KOR	[188]
geoditin A (**196**)/sponge	Terpenoid ^f^	Melanogenesis inhibition	1 μg/mL	cAMP-dependent signaling inhibition	CHN, USA	[189]
gorgosterol (**197**)/soft coral	Terpenoid ^f^	FXR transactivation antagonism	10 μM	Inhibition of OSTα & BSEP genes	ITA	[190]
gracilioether B (**198**)/sponge	Polyketide ^e^	PPARγ binding	5 μM *	Cys285 covalent binding	FRA, ITA	[191]
gracilioether K (**199**)/sponge	Polyketide ^e^	PXR agonistic activity	10 μM *	Binding to LBD by molecular docking	ITA	[192]
herdmanine K (**200**)/ascidian	Alkaloid ^g^	PPAR-γ agonist	1 μg/mL *	mRNAexpression of target genes	S. KOR	[193]
hyrtioreticulin A (**201**)/sponge	Alkaloid ^g^	Ubiquitin-activating enzyme inhibition	2.4 μM	Putative ubiquitin-adenylate intermediate inhibition	IDN, JPN, NLD	[194]
InhVJ protease inhibitor (**202**)/sea anemone	Peptide ^g^	Trypsin and α-chymotrysin inhibition	**	Glu45 involved in InhVJ-trypsin complex	BEL, RUS	[195]
jaspamide (**203**)/sponge	Peptide ^g^	Decreased cardiomyocyte activity and function	1–19 μM *	Kv1.5 channel inhibition	USA	[196]
latonduine A (**204**)/sponge	Alkaloid ^g^	F508del-CTFR correction	1 μM *	PARP-3 inhibition	CAN	[197]
leucettine L41 (**205**)/sponge	Alkaloid ^g^	DYR and CL tyrosine kinase inhibition	21–77 nM	Primary and secondary targets identified	FRA	[169]
manzamine A (**206**)/sponge	Alkaloid ^g^	Cholesterol esters inhibition	4.1 μM	ACAT inhibition	JPN	[198]
nahuoic acid A (**207**)/bacterium	Polyketide ^e^	SETDH inhibition	6.5 μM	Competitive inhibition	PNG, CAN	[199]
namalide (**208**)/sponge	Peptide ^g^	Carbopeptidase A inhibition	0.25 μM	d-Lys presence required for activity	ITA, USA	[200]
ningalins C & D (**209**,**210**)/ascidian	Alkaloid ^g^	CK1δ and GSK3β inhibition	0.2 μM	Binding to ATP binding site	AUS	[201]
octaphlorethol A (**114**)/alga	Polyketide ^e^	Glucose tansporter 4 increase	10 μM *	AKT and AMPK activation	S. KOR	[120]
petrosaspongiolide M (**211**)/sponge	Terpenoid ^f^	Proteasome inhibition	0.085–1.05 μM	Pro-apoptotic bax induction	ITA	[202]
petrosiol A (**212**)/sponge	Polyketide ^e^	PDGF-induced DNA synthesis inhibition	0.73 μM	PDGF receptor-β signaling inhibition	JPN	[203]
phidianidine A (**213**)/mollusc	Alkaloid ^g^	CXCR4 ligand antagonist	<50 μM	CXCL12-dependent DNA synthesis inhibition	ITA	[204]
Poly-APS (**214**)/sponge	Polyketide ^e^	Thoracic aorta contraction inhibition in vitro	<10 μM *	Concentration-dependent LDH release	SVN	[205]
*Pseudoceratina* sp. Dibromotyrosine (**215**)/sponge	Alkaloid ^g^	Apoptosis induction	5 μg/mL	Mitochondrial disfunction	EGY, TWN	[206]
pseudopterosin A (**216**)/soft coral	Terpenoid ^f^	Increased HUVEC proliferation	13 nM	Enhancement potency by HPβCD	USA	[207]
sargachromanol G (**217**)/alga	Terpenoid ^f^	Osteoclastogenesis inhibition	20 Μm *	NF-ĸB phosphorylation of MAPK kinases inhibition	S. KOR	[208]
*S. graminifolium* polysaccharide (**218**)/alga	Polysaccharide ^h^	Improved mitochondrial disfunction and oxidative stress	25 mg/kg ***	Increased activity of antioxidant enzymes	CHN	[209]
*S. patens* phloroglucinol **(219**)/alga	Polyketide ^e^	α-amylase inhibition	3.2 μg/mL	Competitive α-amylase inhibitor	JPN	[210]
*S. xiamenensis* benzopyran (**220**)/bacterium	Mixed biogenesis	Fibrosis inhibition	30 μg/mL *	Anti-proliferation, anti-contractile and anti-adhesion activity	CHN	[211]
theonellasterol (**221**)/sponge	Terpenoid ^f^	Farnesoid receptor transactivation inhibition	50 μM *	SAR showed OH at C-4 and oxidation at C-3 required	ITA, JPN	[212]
toluquinol (**222**)/fungus	Shikimate	Angiogenesis inhibition in vitro and in vivo	2.5 μM *	Cell cycle arrest induction	ESP	[213]
*U. lactuca* fatty acid (**223**)/alga	Polyketide ^e^	ARE activator	10 μg/mL *	Nrf2 transcription factor activation	USA	[214]
alotaketal C (**224**)/sponge	Terpenoid ^f^	cAMP signaling activation	6.5 μM	Undetermined	CAN	[215]
aspergentisyl A(**225**)/fungus	Polyketide ^e^	DPPH radical-scavenging	9.3 μM	Undetermined	CHN	[216]
*A. terreus* butyrolactone (**226**)/fungus	Shikimate	β-glucuronidase inhibition	6.2 μM	Undetermined	LKA, PAK, USA	[217]
caulerpine (**227**)/alga	Alkaloid ^g^	Spasmolytic effect on guinea pig ileum	0.05–0.13 μM	Undetermined	BRA	[218]
conicasterol F (**228**)/sponge	Terpenoid ^f^	FXR antagonism	10 μM *	Undetermined	GBR, ITA	[219]
*D. avara* sesquiterpene (**229**)/sponge	Terpenoid ^f^	FAK, IGF1 & ERBB2 kinase inhibition	1 μg/mL *	Undetermined	DEU, GBR, EGY, SAU	[220]
*D. gigantea* sterols (**230**,**231**)/soft coral	Terpenoid ^f^	Farnesoid receptor transactivation inhibition	14–15 μM	Undetermined	S. KOR	[221]
dysidavarone A (**232**)/sponge	Terpenoid ^f^	PTP1B inhibition	9.98 μM	Undetermined	CHN	[222]
galvaquinone B (**233**)/bacterium	Polyketide ^e^	Epigenetic activity	1.0 μM	Undetermined	USA	[223]
halicloic acids A & B (**234**,**235**)/sponge	Terpenoid ^f^	IDO1 inhibition	10 & 11 μM	Undetermined	CAN, NLD	[224]
isochromophilone XI (**236**)/fungus	Polyketide ^e^	PD4 inhibition	8.3 μM	Undetermined	DEU	[225]
leucettamols A & B (**237**,**238**)/sponge	Terpenoid ^f^	TRPA1 and TRPM8 channel inhibition	4.7–6.4 μM	Undetermined	ITA	[226]
manadosterol A (**239**)/sponge	Terpenoid ^f^	Ubiquitin E2 enzyme UBc13-Uev1A complex inhibition	90 nM	Undetermined	IDN. JPN, NLD	[227]
marilines A_1_ & A_2_ (**240**,**241**)/fungus	Mixed biogenesis	HLE inhibition	0.86 μM	Undetermined	DEU, GRC, PAN	[228]
methyl sarcotroate B (**242**)/soft coral	Terpenoid ^f^	PTP1B inhibition	6.97 μM	Undetermined	CHN	[229]
*P. citrinum* sorbicillinoid (**243**)/fungus	Polyketide ^e^	Antioxidant	30 μM	Undetermined	JPN	[230]
phosphoiodyn A (**244**)/sponge	Polyketide ^e^	hPPARδ inhibition	23.7 nM	Undetermined	AUS, S. KOR	[231]
purpuroines A & D (**245**,**246**)/sponge	Alkaloid ^g^	LCK kinase inhibition	0.94, 2.35 μg/mL	Undetermined	DEU, CHN	[232]
santacruzamate A (**247**)/bacterium	Alkaloid ^g^	HDAC2 inhibition	0.110 nM	Undetermined	PAN, USA	[233]
sarcophytonolide N (**248**)/soft coral	Terpenoid ^f^	PTP1B inhibition	5.9 μM	Undetermined	CHN, ITA	[234]
sargassumol (**249**)/alga	Polyketide ^e^	Antioxidant	47 μM	Undetermined	S. KOR	[235]
sesquibastadin 1 (**250**)/sponge	Alkaloid ^g^	Protein kinases inhibition	0.1–6.5 μM	Undetermined	CHN, DEU	[236]
*S. glaucum* cembranoids (**251**–**253**)/soft coral	Terpenoid ^f^	Cytochrome P450 1A inhibition	12.7–3.7 nM *	Undetermined	EGY, SAU, USA	[237]
symplocin A (**254**)/bacterium	Peptide ^g^	Cathepsin E inhibition	0.3 nM	Undetermined	USA	[238]
tsitsikammamine A derivative (**255**)/sponge	Alkaloid ^g^	IDO1 inhibition	0.9 μM	Undetermined	BEL, FRA	[239]
*V. lanosa* bromophenol (**256**)/alga	Terpenoid ^f^	Biochemical & cellular antioxidant activity	30 μg/mL	Undetermined	NOR	[240]
*X. testudinaria* fatty acid (**257**)/sponge	Polyketide ^e^	Adipogenesis stimulation	2 μM	Undetermined	JPN	[241]

(^a^) **Organism**: *Kingdom Animalia*: soft corals and sea anemone (Phylum Cnidaria), starfish (Phylum Echinodermata), mollusk (Phylum Mollusca); sponge (Phylum Porifera); *Kingdom Plantae:* alga; *Kingdom Monera*: bacterium; (^b^) **IC_50_**: concentration of a compound required for 50% inhibition in vitro; *: estimated IC_50_; **: *K*i 7.4 × 10^−8^ M, and 9.9 × 10^−7^ M, respectively; ***: in vivo study; (^c^) **MMOA**: molecular mechanism of action; (^d^) **Country**: AUS: Australia; BEL: Belgium; BRA: Brazil; CAN: Canada; CHN: China; DEU: Germany; EGY: Egypt; FRA: France; ESP: Spain; GBR: United Kingdom; GRC: Greece; IDN: Indonesia; ITA: Italy; JPN: Japan; LKA: Sri Lanka; NLD: The Netherlands; NOR: Norway; PAN: Panama; PAK: Pakistan; PNG: Papua New Guinea; PYF: French Polynesia; RUS: Russian Federation; SAU: Saudi Arabia; S. KOR: South Korea; SVN: Slovenia; TWN: Taiwan; **Chemistry**: (^e^) Polyketide; (^f^) Terpene; (^g^) Nitrogen-containing compound; (^h^) polysaccharide; **Abbreviations**: ACAT: acyl-CoA:cholesterol acyl-transferase; Akt: protein kinase B; AMPK: AMP-activated protein kinase; ARE: antioxidant-response element; ASIC3: pH-sensitive sodium ion channel 3; CFTR: cystic fibrosis transmembrane conductance regulator; CXCR4: chemokine receptor; CKL: cdc2-like kinase; DYRK: dual-specificity, tyrosine phosphorylation regulated kinase; ERBB2: erb-b2 receptor tyrosine kinase; FAK: focal adhesion kinase; FXR: farnesoid-X-receptor; HDAC: histone deacetylase; HLE: human leukocyte elastase; HUVEC: human umbilical vein endothelial cells; HPβCD: hydroxypropyl-β-cyclodrextrin; IDO1: indoleamine 2, 3 dioxygenase; Kv1.5: Potassium voltage-gated ion channel; LBD: ligand binding domain; LCK: lymphocyte-specific protein tyrosine kinase; IGF1-R: insulin-like growth factor 1 receptor; PDGF: platelet-derived growth factor; PI3K: phosphoinositide 3-kinase; Poly-APS: polymeric 3-alkylpyridinium salts; PARP: poly(ADP-ribose) polymerase; PD4: phosphodiesterase 4; PPARγ: peroxisome proliferator-activated receptor γ; PTP1B: protein tyrosine phosphatase 1B; PXR: pregnane-X-receptor; SETDH: protein methyltransferase SETD8; TRPA1: ankyrin channel; TRPM8: melastin channel.
